# Theta-gamma phase amplitude coupling in a hippocampal CA1 microcircuit

**DOI:** 10.1371/journal.pcbi.1010942

**Published:** 2023-03-23

**Authors:** Adam Ponzi, Salvador Dura-Bernal, Michele Migliore

**Affiliations:** 1 Institute of Biophysics, National Research Council, Palermo, Italy; 2 Department of Physiology and Pharmacology, SUNY Downstate Health Sciences University, Brooklyn, New York, United States of America; 3 Center for Biomedical Imaging and Neuromodulation, The Nathan S. Kline Institute for Psychiatric Research, Organgeburg, New York, United States of America; University of California San Diego, UNITED STATES

## Abstract

Phase amplitude coupling (PAC) between slow and fast oscillations is found throughout the brain and plays important functional roles. Its neural origin remains unclear. Experimental findings are often puzzling and sometimes contradictory. Most computational models rely on pairs of pacemaker neurons or neural populations tuned at different frequencies to produce PAC. Here, using a data-driven model of a hippocampal microcircuit, we demonstrate that PAC can naturally emerge from a single feedback mechanism involving an inhibitory and excitatory neuron population, which interplay to generate theta frequency periodic bursts of higher frequency gamma. The model suggests the conditions under which a CA1 microcircuit can operate to elicit theta-gamma PAC, and highlights the modulatory role of OLM and PVBC cells, recurrent connectivity, and short term synaptic plasticity. Surprisingly, the results suggest the experimentally testable prediction that the generation of the slow population oscillation requires the fast one and cannot occur without it.

## Author summary

Oscillations are a prominent and ubiquitous feature of brain operations. They are found across multiple different animal species and with a variety of recording techniques from small-scale microelectrodes up to large-scale EEG and fMRI. Oscillations appear in a range of discrete frequencies, such as theta (3-8 Hz) and gamma (25-100 Hz) in the hippocampus. Notably, these oscillations are found to be coupled whereby the phase of the slower oscillation modulates the amplitude of the faster oscillation. The strength of this ‘phase-amplitude coupling’ is itself found to depend on the animals cognitive state and ongoing behaviour, and is therefore thought to play an important functional role. Despite the importance of this phenomenon, its biophysical origin and the reasons for its ubiquity across brain regions and species remain unclear. Here, using a data-driven physiologically detailed computational model of hippocampal CA1, we show how a basic microcircuit can autonomously generate both theta and gamma which are phase-amplitude coupled. The results suggests that the underlying dynamical mechanism is very general, and will be relevant in multiple different neural settings.

## Introduction

Phase amplitude coupling (PAC), whereby the phase of a slow neuronal oscillation modulates the amplitude of a fast oscillation is found in many brain areas in multiple species with a variety of neuronal recording techniques and plays important functional roles [1–5]. In the hippocampus the phase of a 3 to 10 Hz theta rhythm modulates the amplitude of a faster gamma rhythm above 30 Hz [5–11]. Different types of theta [12–15] and gamma [10, 16–19] can be transmitted from various brain areas but they can also both be generated intrinsically in the hippocampus [14, 15, 20–28]. Multiple neuronal types are involved in intrinsic hippocampal oscillogenesis and theta-gamma PAC, but the underlying network and cellular mechanisms are debated [29].

Several general frameworks for PAC generation have been proposed and reviewed in detail in [2, 29–31]. Most models include two subsystems, either neuronal populations or different cell types, which include different timescale components and therefore oscillate at different frequencies. In the hippocampus most models generate theta-gamma PAC [32–39] by including a cell model which intrinsically fires at theta frequency and another cell population which generates gamma frequencies by pyramidal-interneuron gamma (PING) or interneuron-gamma (ING) [40–50], or alternatively two subpopulations which generate oscillations at different frequencies (by PING or ING like mechanisms). PAC occurs when the two populations interact either directly or through a third population of pyramidal (PYR) cells. However a recent paper has proposed a different model of how slow (delta or theta) and fast (gamma) frequencies interact using a network of coupled intrinsically bursting cells [51].

Basket cells expressing parvalbumin (PVBC) are often thought to be the main contributors to gamma [24, 52], because they show enhanced responses to gamma frequency inputs [53, 54] and target PYR proximal dendrites to generate a rapid and strong postsynaptic response. Theta, on the other hand, is often thought to be generated by the intrinsic pacemaking properties of GABAergic oriens-lacunosum/moleculare (OLM) interneurons expressing somatostatin (SST) [32, 33, 53, 55], which inhibit PYR distal dendrites [56]. However, several recent studies have challenged these circuit architectures for theta-gamma PAC and, on the contrary, suggest that theta and gamma emerge as a single unitary process and cannot be dissociated [57]. In particular gamma and theta frequencies [10] and powers [57] strongly covary during behaviour. This observation may be more difficult to explain if theta and gamma arise from distinct circuit or biophysical mechanisms since one may expect to find theta and gamma powers and frequencies varying independently under certain circumstances.

OLMs are also found to have a more complex role in both theta and gamma. Although OLM activity is modulated by theta rhythms [12, 23, 24, 58–60] and they spike at theta frequencies [20, 53, 55, 61–63], they are not able to generate theta intrinsically under *in-vivo*-like conditions [54], and they do not seem to show strong pacemaker properties when driven with unmodulated synaptic input [64], even though they are able to follow theta modulated synaptic input effectively [62, 64]. Despite these findings, constant optogenetic activation of OLMs in running mice induced theta-gamma PAC at theta and gamma frequencies different to those present without the stimulation [15]. Strikingly PYRs were phase locked to the induced theta, while OLM stimulation did not elicit theta at all when PYRs were inhibited. In a CA1 slice, optogenetic stimulation of PYRs at theta frequency induced robust gamma oscillations, which were phaseamplitude coupled to the theta stimulation [25]. The gamma oscillations were dependent on both AMPA and GABA, suggesting a PING like mechanism which could be evoked by theta stimulation [65]. Intriguingly, the power of induced gamma was also found to have a peak at a particular frequency of the driving theta, suggesting that gamma power itself varies with its own intrinsic timescale. Besides theta, other studies in different brain areas have found a role for SSTs in gamma oscillations [66–68]. These studies implicate a feedback OLM-PYR circuit in the generation of both theta and gamma and theta-gamma PAC but the mechanism is unknown.

Here we describe how PAC arises in a detailed *data-driven* CA1 microcircuit model, composed of morphologically detailed PYR, OLM and PVBC cell models previously optimized against a number of experimental studies [73], and with synaptic facilitation and depression properties also validated against data [69, 70]. We find that theta and slow-gamma PAC emerged without fine tuning of parameters mediated by a single feedback circuit of OLM and PYR cells. We find that the optimized OLM cells have an intrinsic tendency to irregularly stutter fire gamma frequency bursts around theta frequencies. When they are coupled via PYR cells a PING type interaction is produced which not only synchronizes them at gamma frequencies but also produces a robust population oscillation at theta frequencies, and this is strongly facilitated by OLM-PYR STP. This relatively simple mechanism, akin to that described in [51], may explain the ubiquity of PAC in the brain, sheds light on multiple empirical observations, and suggests experimentally testable predictions.

## Results

### Model validation

**Fig 1:**
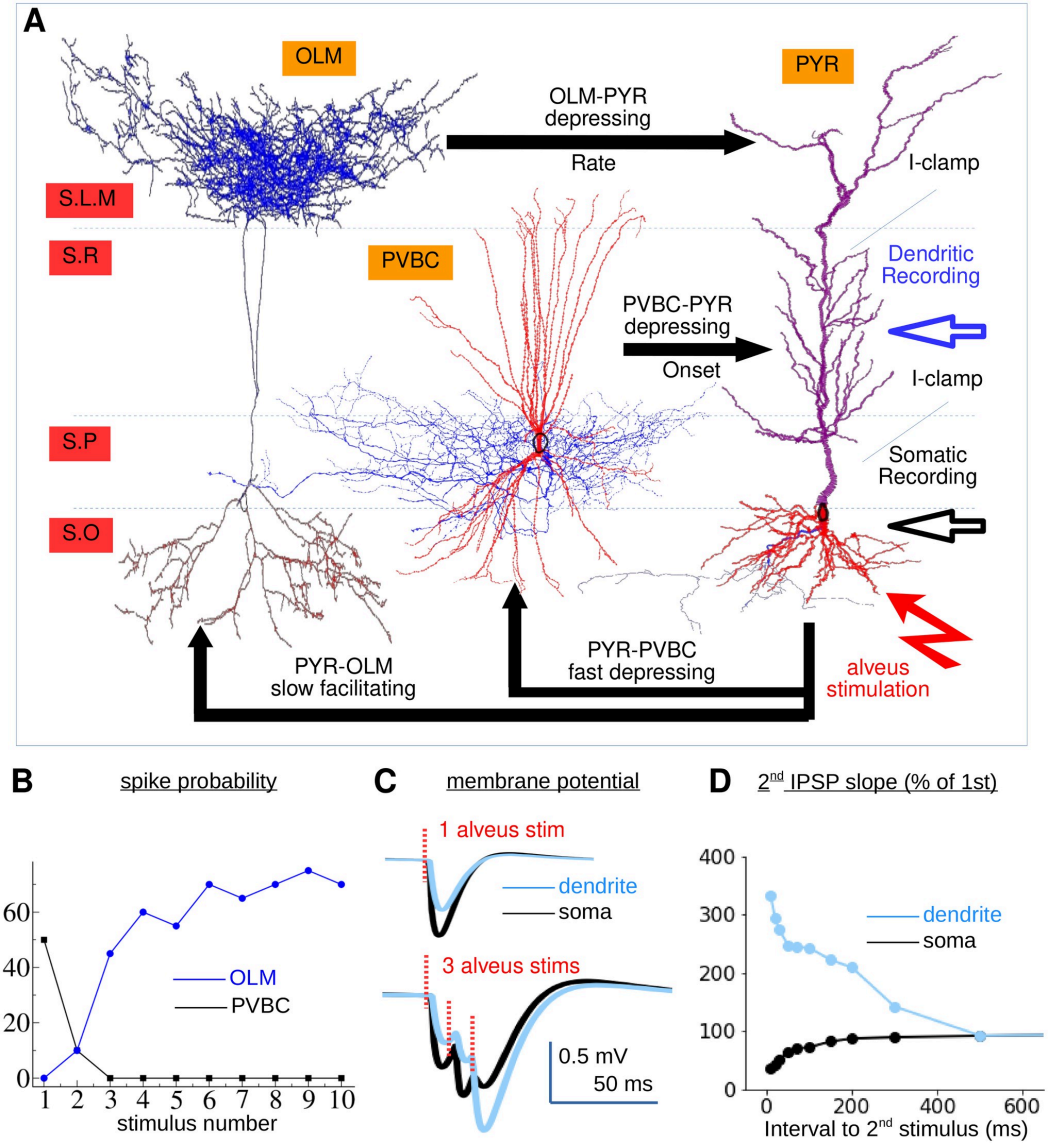
Model validation for comparison with experimental results in [69]. **(A)** *Schematic of microcircuit configuration. PVBC cells target the PYR soma or dendrites within about 50 microns of the soma. OLM cells on the other hand target the PYR distal apical dendrites at least 250 microns from the soma. Cells are connected to random locations within the appropriate dendritic domains using the appropriate number of synapses per connection estimated from studies* [70]. *Feedforward connections are activated by alveus stimuli. Recording locations are indicated at PYR soma and at locations in the proximal region of the apical dendrite as in* [69]. *As in* [69], *the PYR cell was current clamped at the soma and at several places in the apical dendrite to amplify IPSPs*. **(B)** *PVBC and OLM (see key) spiking probability versus stimulus number. Values shown are averages across eighty cells with different random synaptic conductance parameters and alveus stimulus locations*. **(C)** *Membrane potential traces in the dendrite and the soma when the circuit is activated with a single alveus stimulus and three successive alveus stimuli at 100 Hz. Note that the dip in the experimental traces following each alveus stimulus is an artifact which should be ignored*. **(D)** *Dependence of successive IPSP size on interstimulus interval*. **(C,D)** *The GABA decay timescale from OLM to PYR cells used here is 18 ms* [56, 71, 72].

In order to understand the origin of CA1 theta-gamma PAC it is important to use a CA1 circuit model which fits the known physiogical empirical data as closely as possible. We used multicompartment cell models with reconstructed dendritic morphologies which had been previously optimized to faithfully reproduce multiple characteristics of CA1 cell spiking activity and synaptic properties [70, 73]. A schematic representation of the circuit and its wiring properties is illustrated in Fig 1A. Implementation details can be found in *Methods*.

The microcircuit included PYR, PVBC and OLM cells since these are most strongly implicated in the generation of theta-gamma PAC. There is a specific set of experimental findings suggesting how these cells may interplay during alveus stimulation [69]. We used these data as a reference to constrain the model parameters.

The model findings on the interneurons spiking probability, as a function of the number of 50 Hz presynaptic alveus stimuli, is shown in Fig 1B. The model is in good agreement with the experimental findings described in [69]. PVBC spiking probability, Fig 1B (black), was about 50% on the first alveus stimulus and decayed to zero after about three stimuli in both the model, Fig 1B (black), and the experimental data (Fig 3D of [69]). On the other hand OLM spiking probability was zero on the first alveus stimuli and increased to around 40% by the third stimulus in both the model, Fig 1B (blue), and the experimental data, (Fig 3D of [69]).

To validate synaptic connections against experimental dendritic and somatic recordings, we connected a small circuit as in Fig 1A, and simultaneously activated the interneurons with alveus stimuli as described in [69]. Typical results are shown in Fig 1C. In good qualitative agreement with experiments (Fig 1B of [69]), we found that with a single alveus stimulus IPSPs recorded in the PYR apical dendrite were smaller than in the soma, Fig 1C (top). However when three alveus stimuli at 100 Hz were applied the first two dendritic IPSPs were again smaller than the somatic ones but, in contrast, the third dendritic IPSP was much larger than the somatic one, Fig 1C (bottom).

These two results highlight this circuit operation: on the first two alveus stimuli PVBC cells spike, but OLMs do not. Since the PVBC cells target the PYR dendrites close to the soma this generates a large IPSP at the soma and a smaller one in the dendrites by backpropagation. On the third alveus stimulus however the OLM cells spike and the PVBC cells do not. The OLM cells generate a large IPSP in the distal apical dendrites which propagates towards the soma, generating a large IPSP in the proximal apical dendrites and a small IPSP in the soma.

The dependence of this effect on the time interval between alveus stimuli was also examined experimentally in [69] by comparing the slope of the onset of the second IPSP, as a proxy for the second IPSP size, with that of the first IPSP. We also calculated this quantity as a final validation of the full circuit, Fig 1D. For small intervals the second dendritic IPSP is much larger than the first, Fig 1D (blue) while the opposite is true for the somatic IPSP, Fig 1D (black). This effect decays with increasing inter-stimulus interval in excellent agreement with the experimental findings (Fig 1G of [69]).

### Theta slow-gamma PAC emerges in a network

**Fig 2:**
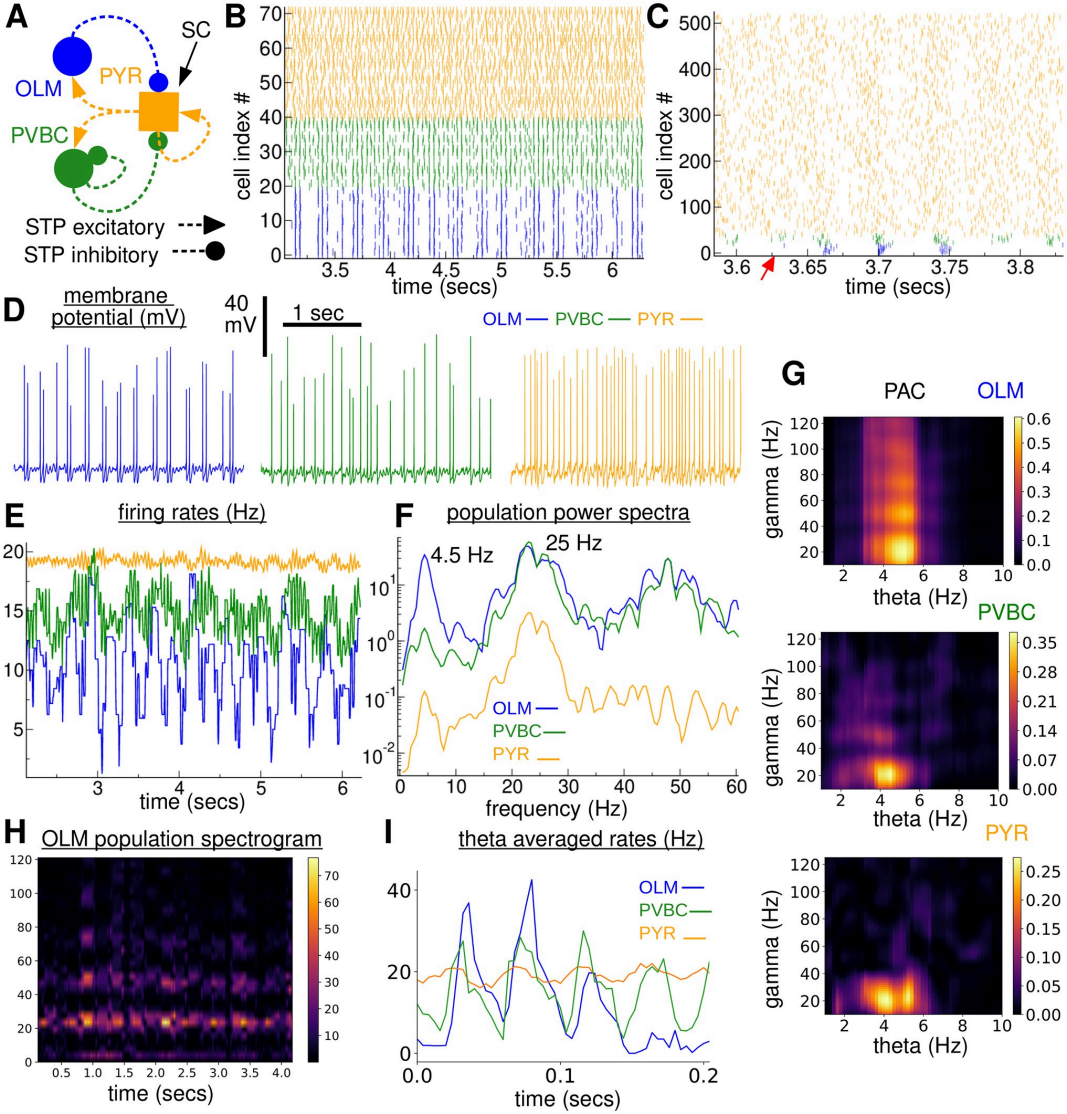
TSGPAC in a 480 PYR, 20 OLM, 20 PVBC cell network simulation of the ‘full model’, (FM). **(A)** *Schematic of the circuit*. **(B)** *Raster plot showing all the OLM and PVBC but only 32 of the PYR cells*. **(C)** *Smaller time segment raster plot showing all 480 PYR. Red arrow indicates onset of a theta burst*. **(b,c)** *OLM (blue), PVBC (green), PYR (orange)*. **D** *Example simultaneously recorded membrane potential traces. (left) OLM, (middle) PVBC, (right) PYR*. **(E)** *Population firing rate time series*. **(F)** *Population activity power spectra for the three cell populations, log y-axis, (blue (OLM), green (PVBC), orange (PYR))*. **(G)** *PAC comodulograms for (top) OLM, (middle) PVBC and (bottom) PYR cell population activity*. **(H)** *Spectrogram for the OLM population activity*. **(I)** *Theta cycle averaged population firing rates for the three cell populations*.

We next investigated how a network of PYR, PVBC and OLM cells behaves when driven by excitatory input from the Schaffer collaterals (SC). We constructed a biologically faithful network (see Methods), which we denote the ‘full model’ (FM), (to distinguish from simpler models described below), including all connections between PYR and interneurons as well as recurrent PYR to PYR and PVBC to PVBC connections, Fig 2A. All connections include STP validated above against [69], or taken directly from [70]. The typical behaviour of an example simulation with 20 OLM, 20 PVBC and 480 PYR cells is shown in Fig 2. Strikingly we found theta slow-gamma PAC (TSGPAC) emerged without fine tuning of parameters.

A clear oscillation at around 4.5 Hz low theta frequency can be seen in the OLM cell population in the spike raster plot section, Fig 2B (blue). OLM cells seem to fire theta frequency bursts of two or three higher frequency spikes around slow gamma frequency, ∼ 25 Hz, which are synchronized across cells. These OLM bursts are separated by periods of silence with occasional spikes. This burst firing activity is also evident in the membrane potential trace from an exemplar OLM cell, Fig 2D (blue). Coincident periods of 25 Hz synchronized spiking are also visible in the PVBC and PYR spiking activity in the raster plot magnification, Fig 2C (green, orange), although, in contrast to the OLM cells, between synchronized bursts the PVBC and PYR activity appears to consist of irregular spiking, incoherent across cells, Fig 2B (green, orange). Both theta and slow gamma rate modulations can be seen in the cell population firing rate time series, Fig 2E, for all three cell populations and this activity is confirmed in the population activity power spectra, Fig 2F, which show peaks at 4.5 Hz theta frequency and 25 Hz slow gamma frequency for all three populations, but most strongly in the OLM population activity (blue). Persistent oscillations are also clear in the OLM population spectrogram, Fig 2H, as horizontal bands around 25 Hz and at higher harmonics, which also show theta frequency amplitude modulation.

Thus the network seems to generate persistent oscillations at two distinct frequencies. To investigate if these two oscillations are coupled we calculated PAC comodulograms, Fig 2G. We found strong TSGPAC in all populations extending from around 4 Hz to 6 Hz in the theta phase frequency and centered on about 25 Hz in the slow gamma amplitude frequency. The OLM comodulogram also shows the 50 and 75 Hz harmonics. To examine the relationship of gamma to theta oscillations in more detail we computed *theta-averaged* activity profiles, Fig 2I. Here population activity is averaged across successive theta cycles obtained from band-passing the activity at the peak theta frequency (see Methods). We found that cell population firing rates were strongly modulated by theta phase, Fig 2I. The amplitude of gamma oscillations is also strongly modulated by theta phase. A strong slow-gamma component appears in all three populations when the OLM firing rate increases at a particular phase of the theta cycle. On each gamma cycle a peak appears first in the PYR population followed by a sharper peak in the PVBC activity and finally the OLM activity peaks on the descending phase of the PYR gamma.

### The dependence of TSGPAC on SC input level

**Fig 3:**
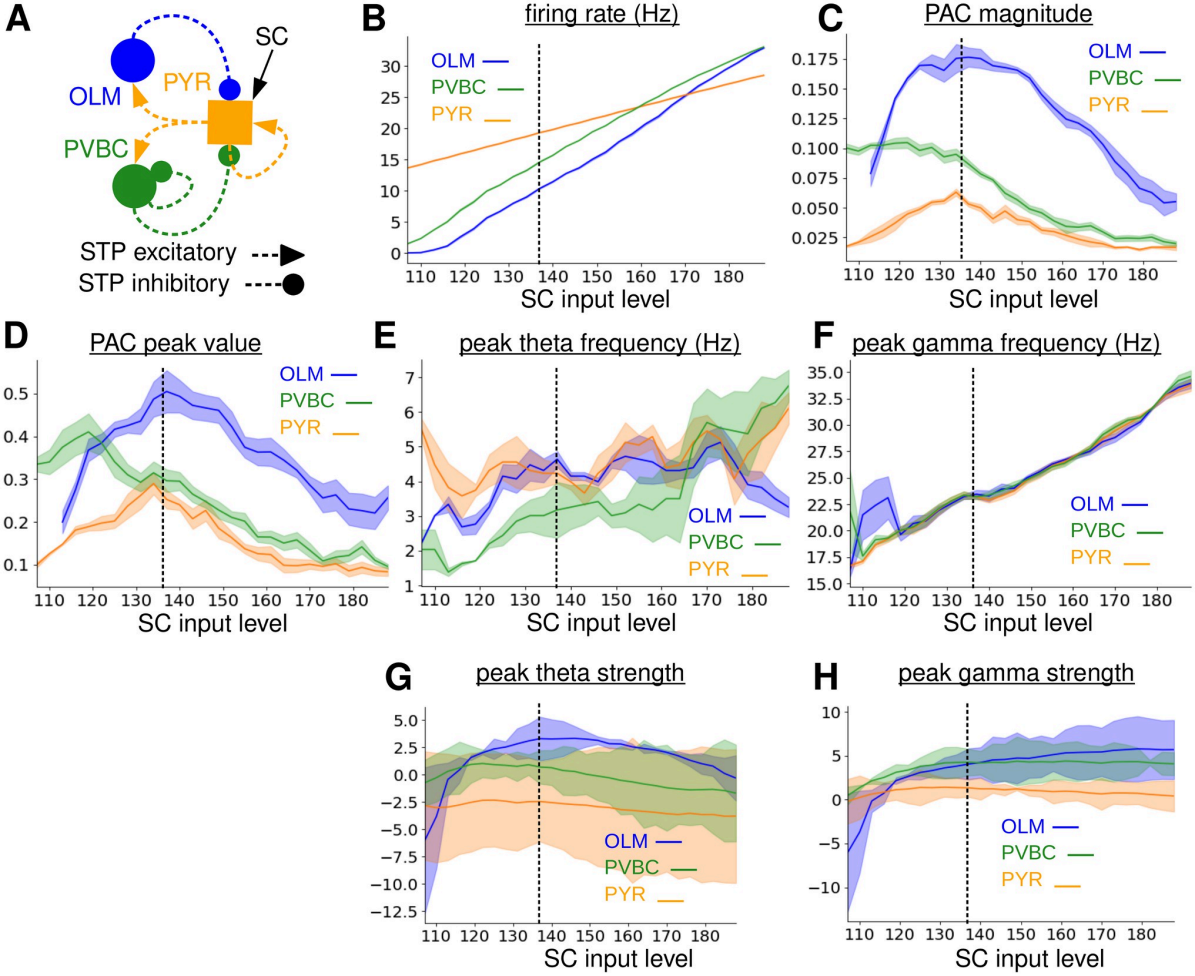
Dependence of TSGPAC on SCIL in the 480 PYR, 20 OLM, 20 PVBC cell full model, (FM). **(A)** *Schematic of the circuit*. **(B)** *Population firing rates*. **(C)** *Total PAC magnitude in the theta, 1-12 Hz, band and slow-gamma, 12-50 Hz, band*. **(D)** *Peak PAC value*. **(E)** *Peak theta frequency. Location of power spectrum peak in 1-8 Hz band*. **F** *Peak gamma frequency. Location of power spectrum peak in 15-50 Hz band*. **(G)** *Theta strength. Log of theta band power spectrum peak size. **(H)** Gamma strength. Log of gamma band power spectrum peak size*. **(B-H)** *Shaded bands show SEM in the plotted values across two identical simulations with random configurations. Dashed vertical line indicates the simulation shown in* Fig 2.

The strength and frequencies of PAC in CA1 are known to depend on the cognitive task and behaviour the animal is engaged in. Thus PAC properties may depend on the strength of driving excitation from the Schaffer collaterals. To investigate this we varied the excitatory driving to the 480 cell PYR population from CA3 by increasing the number of active independent (Poisson spiking) SC inputs to each PYR cell, denoted ‘SC input level’, (SCIL). This resulted in a variation between about 13 Hz and 26 Hz in the PYR firing rate, Fig 3B (orange). We found that strong TSGPAC appeared in the OLM population, Fig 3C (blue), soon after the OLM cells cross firing threshold at around SCIL 120, Fig 3B (blue). Above this SCIL, mean TSGPAC magnitude, Fig 3C, and peak TSGPAC magnitude, Fig 3D, strongly increased with SCIL for the OLM population (blue), and more weakly for the PYR population (orange), peaking around SCIL 140. The PVBC population (green) also shows TSGPAC in this SCIL range but also at lower SCIL, albeit much more weakly than the OLM population. The dashed vertical line in Fig 3B–3H indicates the simulation illustrated in Fig 2.

We also investigated how the theta and gamma frequencies depend on the SCIL. The lines in Fig 3E and 3F show the locations of the peak frequencies in the theta (1-8 Hz) and slow gamma (15–50 Hz), bands, respectively, in the population power spectrum for the three cell types. The presence of a peak does not necessarily mean it is strong, so peak strengths for theta, Fig 3G, and slow gamma, Fig 3H are also shown. We find the OLM population has a strong peak between about 4 and 5 Hz in the theta range, Fig 3E and 3G (blue), between about SCIL 120 and SCIL 170, but the PYR and PVBC theta peaks are weaker. On the other hand power spectra of all three cell populations have a strong peak in the 15-50 Hz slow-gamma band above about SCIL 120, Fig 3H, with frequency monotonically increasing between about 20 Hz and 32 Hz, Fig 3F. PYR slow-gamma power, Fig 3H (orange), is weaker than OLM and PVBC slow-gamma power because PYR activity is a bit sparser, whereby individual PYR do not spike on each gamma cycle, and because theta bursts are separated by periods of incoherent irregular spiking, Fig 2B and 2C (orange).

These results demonstrate that in a network wired the same way as the real system, strong robust TSGPAC at physiologically observed frequencies naturally emerges for a quite large range of SCIL. The fact that theta power, Figs 2F and 3G (blue) and TSGPAC, Figs 2G, 3C and 3D (blue), are strongest in the OLM population, suggests that theta and TSGPAC are being generated by theta periodic bursts of slow-gamma oscillations in the OLM population. To clarify how OLM cell intrinsic spiking properties, network reverberation mechanisms and synaptic STP interplay to generate TSGPAC we first modified the OLM-PYR feedback circuit.

### OLM cells generate weak theta-gamma PAC intrinsically

We first considered that TSGPAC might be generated simply by the isolated OLM cells themselves. In fact, while we were not able to induce OLM cells to show bursting when driven by somatic current injection, they can show both theta and slow gamma themselves, albeit very weakly, when driven by the 480 spiking PYR cells in a purely feedforward way. To demonstrate this we performed simulations of a modified full model (FM) where the OLM-PYR feedback connections are removed, denoted FMx.

**Fig 4:**
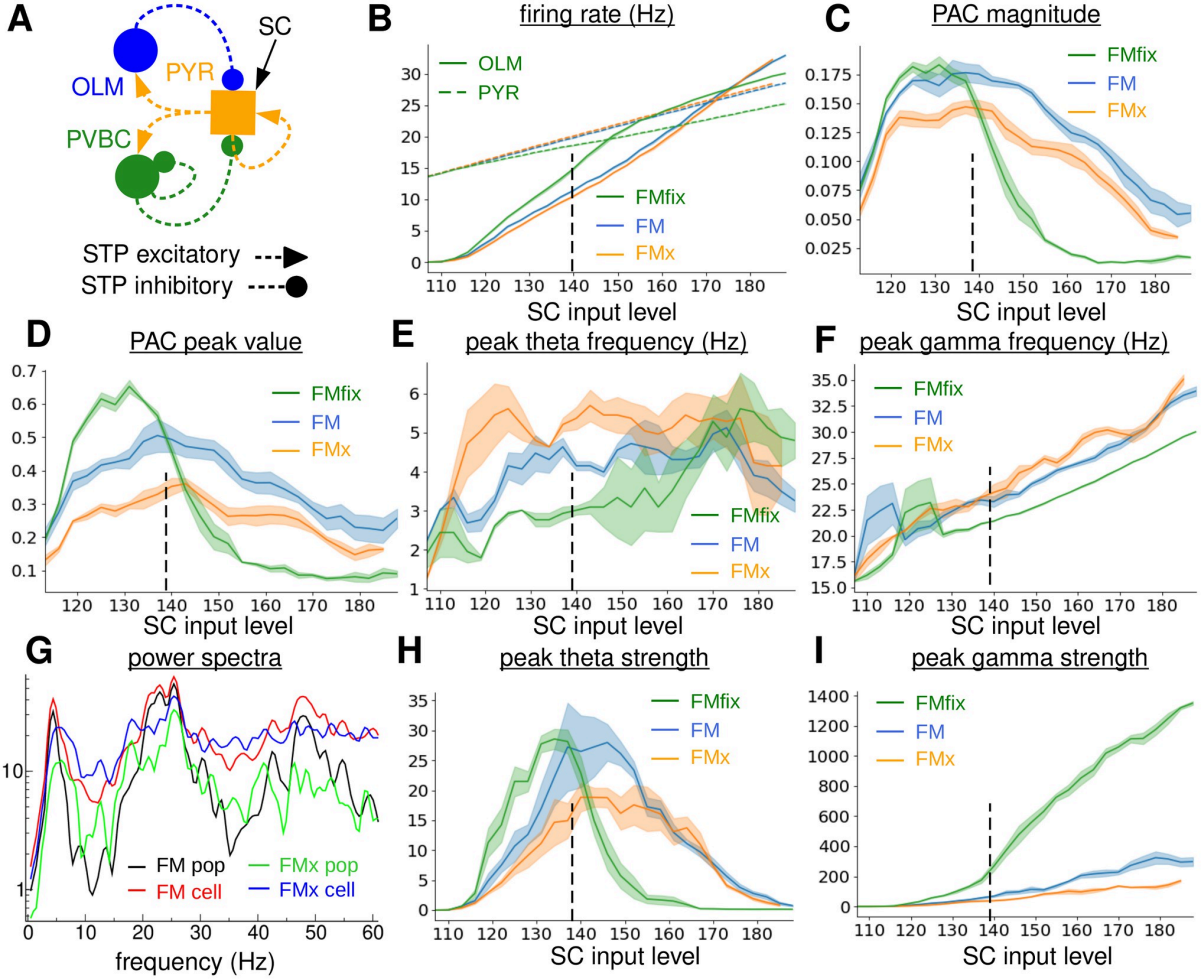
Dependence of TSGPAC on SCIL in the 480 PYR, 20 PVBC, 20 OLM full model with normal depressing STP OLM-PYR connections, denoted ‘FM’ (blue), no OLM-PYR connections, denoted, ‘FMx’, (orange), fixed strength OLM-PYR connections, denoted ‘FMfix’, (green). **(A)** *Schematic of the network with facilitating/suppressing connections*. **(B)** *OLM (solid) and PYR (dashed) firing rates*. **(C)** *Total OLM population PAC magnitude in the theta, 1-12 Hz, band and slow-gamma, 12-50 Hz, band*. **(D)** *OLM population PAC peak value*. **(E)** *OLM population peak theta frequency. Location of power spectrum peak in 1-8 Hz band*. **(F)** *OLM population peak gamma frequency. Location of power spectrum peak in 15-50 Hz band*. **G** *OLM power spectra for a particular simulation at SCIL 140, indicated by the vertical dashed line in (b-f,h,i). (Black, green) population activity power spectrum for (black) the full model, FM, (green) the model without feedback OLM-PYR connections, FMx. (Red, blue) average single cell activity power spectrum for (red) the full model, FM, (blue) the model without feedback OLM-PYR connections, FMx*. **(H)** *OLM population theta strength. Theta band power spectrum peak size*. **(I)** *OLM population gamma strength. Gamma band power spectrum peak size*. **(B-F,H,I)** *Shaded bands show SEM in the plotted values across two simulations with random configurations. Dashed vertical line indicates the simulation shown in G*.

Even without OLM-PYR feedback in the FMx OLM population activity shows a peak in TSGPAC, Fig 4C and 4D (orange), as SCIL is increased, similar to, but much weaker than, that shown by the OLM population in the FM, which is reproduced in Fig 4C and 4D (blue) for direct comparison. OLM theta strength, Fig 4H (orange), also shows a peak at an intermediate level of SCIL in the FMx, but it is again much weaker than in the FM, reproduced in Fig 4H (blue). Slow-gamma strength increases monotonically, Fig 4I (orange), but again is much weaker than in the FM, Fig 4I (blue).

Theta and gamma frequencies in the FMx, Fig 4E and 4F (orange), are somewhat higher than their FM counterparts, Fig 4E and 4F, (blue). Exemplar OLM power spectra from simulations with and without OLM-PYR feedback at SCIL 140 are compared in Fig 4G. As described above the OLM population power spectrum, Fig 4G (black), for the FM shows strong peaks at 4.5 Hz theta and around 25 Hz gamma. However the corresponding OLM population power spectrum without OLM-PYR feedback in the FMx, Fig 4G (green), also shows weak peaks at around 6 Hz theta and 28 Hz gamma. These peaks become quite a lot stronger when the power spectra are calculated from individual cells and then averaged, Fig 4G (blue), instead of from the population activity, but the peaks remain weaker than power spectra calculated from individual cells in the FM, Fig 4G (red).

These observations suggest that OLM cells generate theta, Fig 4H (orange), and gamma, Fig 4I (orange), activity themselves when they are driven into a regime a few Hz above threshold by many spiking PYR cells, but when too strongly excited they only spike at gradually increasing slow gamma frequencies, Fig 4I (orange). However they are not synchronized without the OLM-PYR feedback and population theta and gamma are therefore weak. OLM-PYR feedback therefore provides a mechanism for amplification and synchronization of theta and gamma intrinsically generated by OLM cells when they are in a excitation regime a few Hz above threshold.

### The role of OLM-PYR synaptic depression in TSGPAC

Given the importance of the OLM-PYR feedback in the generation of strong TSGPAC and the fact that OLM firing rates are strongly varying, Fig 2E (blue), we expected that OLM activity dependent OLM-PYR short term synaptic depression should play a role in TSGPAC. Surprising we found that OLM-PYR STP was not necessary for strong TSGPAC. However, removal of OLM-PYR synaptic depression in the full model, denoted FMfix, Fig 4(green), does have several effects. First, the SCIL window where theta, Fig 4H (green), and TSGPAC, Fig 4C and 4D (green), are strong is restricted to a narrower range of lower SCIL than the FM, Fig 4C, 4D and 4H (blue). Second, the theta, Fig 4E (green) and gamma, Fig 4F (green), frequencies are reduced compared to their FM values, Fig 4E and 4F (blue). Third the gamma strength is strongly increased, Fig 4I (green). Fourth the OLM firing rate, Fig 4B (solid green), increases more rapidly at lower SCIL and shows a cross-over to a slower rate of increase around SCIL 150. Similarly the PYR firing rate, Fig 4B (dashed green), increases more slowly. Thus we find that while not strictly necessary, OLM-PYR synaptic depression does make TSGPAC more robust across a wider range of SCIL.

### Network generated PING-like reverberation is necessary for synchronization across OLM cells

**Fig 5:**
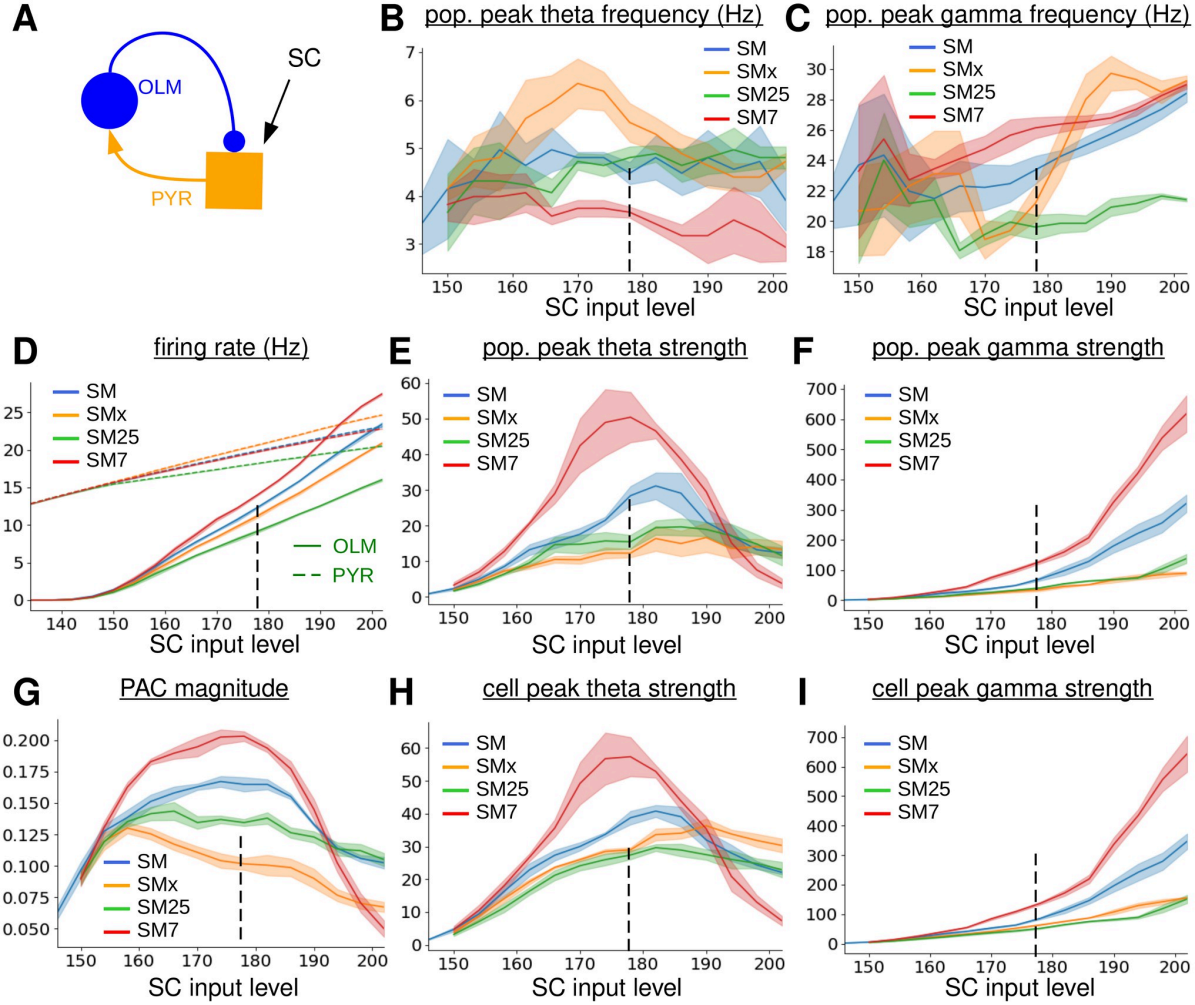
Dependence of TSGPAC on SCIL in the 120 PYR, 10 OLM simplified feedback circuit model with normal strength OLM-PYR synapses, denoted ‘SM’ (blue), no OLM-PYR connections, denoted ‘SMx’, (orange), strong OLM-PYR synapses and 25 ms GABA decay timescale, denoted ‘SM25’, (red) and strong OLM-PYR synapses and 7 ms GABA decay timescale, denoted ‘SM7’, (green). **(A)** *Schematic of the simplified circuit model*. **(B)** *OLM population peak theta frequency. Location of power spectrum peak in 1-8 Hz band*. **(C)** *OLM population peak gamma frequency. Location of power spectrum peak in 15-50 Hz band*. **(D)** *OLM (solid) and PYR (dashed) firing rates*. **(E)** *OLM population theta strength. Theta band power spectrum peak size*. **(F)** *OLM population gamma strength. Gamma band power spectrum peak size*. **(G)** *OLM population total PAC magnitude in the theta, 1-12 Hz, band and slow-gamma, 12-50 Hz, band*. **(H)** *OLM single cell theta strength averaged across cells. Theta band power spectrum peak size*. **(I)** *OLM single cell slow gamma strength averaged across cells. Slow gamma band power spectrum peak size*. **(B-I)** *Shaded bands show SEM in the plotted values across two simulations with random configurations. Dashed vertical line indicates the simulation described in* Fig 6.

To understand these STP effects and investigate TSGPAC more easily we made a greatly simplified model, denoted ‘SM’, containing only the necessary model components. The SM included only OLM and PYR cells in a feedback circuit without PVBC cells or recurrent connectivity amongst the PYR cells, Fig 5A, (see Methods) and without OLM-PYR depressing STP. However we retained PYR-OLM facilitating STP to facilitate comparison with the FM model. In fact since the PYR firing rate does not vary strongly, Fig 2E (orange), removing PYR-OLM facilitation does not have a strong effect on TSGPAC properties providing the PYR-OLM synaptic strength is increased to compensate for the absence of facilitation (see Discussion). To reduce computational cost we also decreased the network size to 120 PYR cells connected to OLMs with four times the quantity of synapses as the FM (which includes 480 PYR), and 10 OLM cells connected to PYR with twice the quantity of synapses as the FM (which includes 20 OLM). We also reduced the OLM-PYR GABA decay timescale since it turns out that this makes TSGPAC stronger (see below). The GABA decay timescale for the OLM to PYR synapses used in the FM, and in the validation against [69], is 18 ms [71, 72]. In the SM we reduced this to 11.8 ms, [74]. Both values have been used in previous modeling studies [71, 74] (see Discussion).

**Fig 6:**
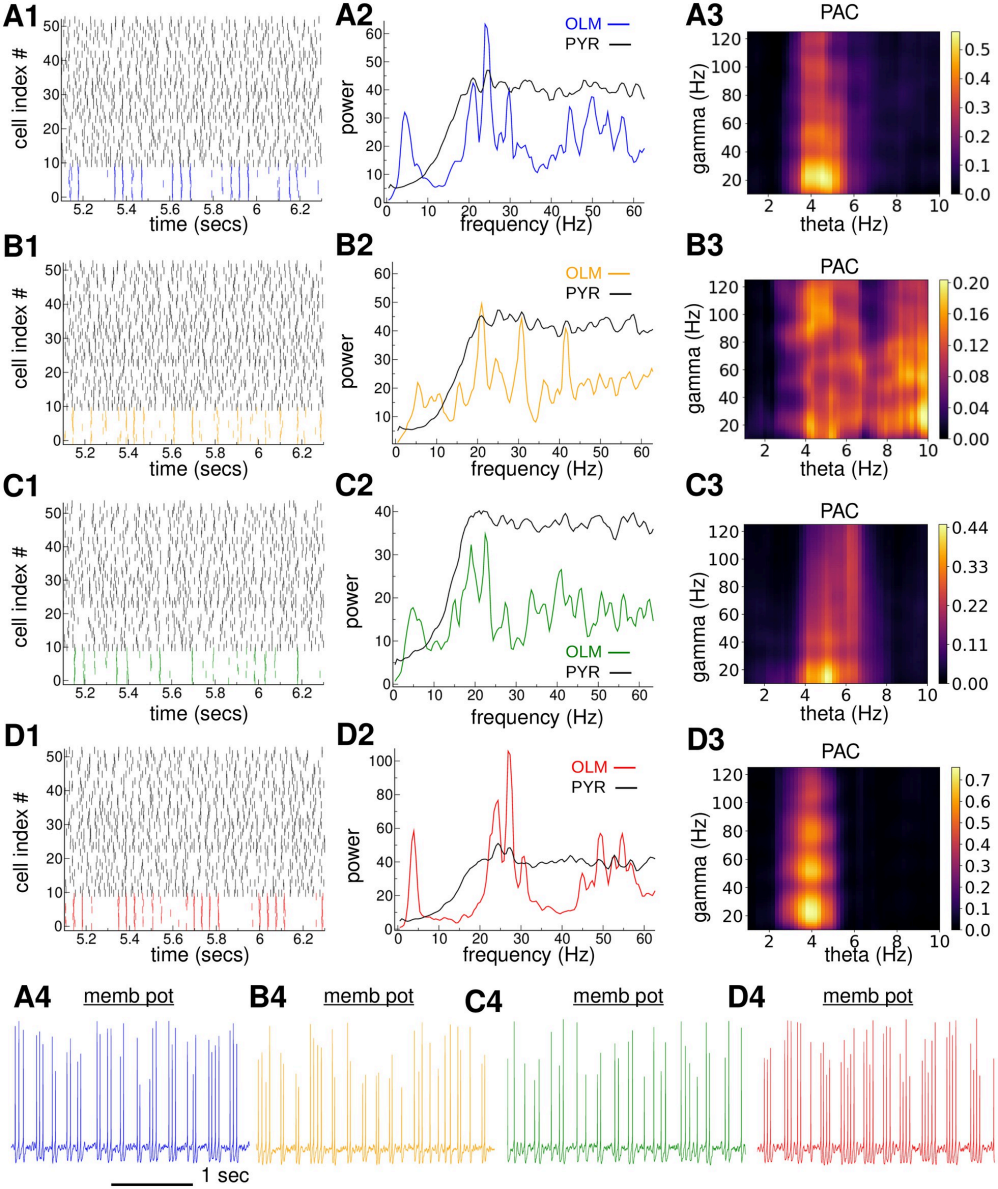
*Exemplar simulation of the 120 PYR,10 OLM simplified feedback circuit model at SCIL 178 (indicated by dashed vertical line in* Fig 5 *) with* **(A1-A4)** *normal strength OLM-PYR synapses, denoted ‘SM’ (blue)*, **(B1-B4)** *no OLM-PYR connections, denoted ‘SMx’, (orange)*, **(C1-C4)** *strong OLM-PYR synapses and 7 ms GABA decay timescale, denoted ‘SM7’, (green) and* **(D1-D4)** *strong OLM-PYR synapses and 25 ms GABA decay timescale, denoted ‘SM25’, (red)*. **(A1-D1)** *Raster plot segment showing all 10 OLM (coloured) and 42 of the 120 PYR (black)*. **(A2-D2)** *Average individual cell power spectrum for OLMs (coloured) and PYRs (black)*. **(A3-D3)** *OLM population activity PAC comodulogram*. **(A4-D4)** *Three second membrane potential traces from examplar OLM cells for each model, ‘SM’ (blue), ‘SMx’, (orange), ‘SM7’, (green) ‘SM25’, (red)*.

The SM too shows a peak in OLM TSGPAC, Fig 5G (blue), and OLM population theta power, Fig 5E (blue) as SCIL is varied. The theta and gamma frequencies are similar to the full model, around 5 Hz, Fig 5B (blue), and 23 Hz, Fig 5C (blue), respectively. Thus strong TSGPAC can be generated by a simple OLM-PYR feedback circuit without STP. An exemplar simulation of the SM at SCIL 178 near the TSGPAC peak, indicated by the dashed line in Fig 5B–5I is shown in Fig 6A. The SM raster plot, Fig 6A1, shows bursts of three or four spikes synchronized across the OLMs (blue) separated by silent periods, similar to the behaviour found for the full network model, Fig 2B. An OLM membrane potential trace from the same simulation, Fig 6A4, also shows this clustered spiking activity. The approximately 5 Hz theta burst frequency and the 23 Hz spiking frequency are evident in the average individual OLM cell power spectrum, Fig 6A2 (blue), and in the OLM population activity PAC comodulogram, Fig 6A3.

We also investigated what happens in the SM when OLM-PYR feedback is removed, denoted SMx, in the same way as described above for the FMx, Fig 4. As in the FMx, OLM TSGPAC, Fig 5G (orange), and OLM population gamma strength, Fig 5F (orange), are strongly reduced, and like in the FMx a peak in population theta power remains, Fig 5E (orange), although it is much weaker than the SM peak, Fig 5E (blue). Despite the fact that the OLM cells are not connected, synchronous events can be seen in their activity in the exemplar raster plot, Fig 6B1 (orange). These occur because, as described above, the individual cells do weakly generate theta and gamma activity, which can be seen as weak peaks in the OLM single cell power spectrum, Fig 6B2, and because they receive common driving input from the PYR population. These frequencies appear as a tendency for irregular gamma frequency stutter bursting around theta frequencies in a typical OLM membrane potential trace from the same simulation, Fig 6B4. The OLM PAC comodulogram, Fig 6B3, does not show strong modulation at theta or gamma, however, because it is constructed from the OLM population activity.

These observations from this simple feedback circuit suggest the following mechanism for TSGPAC here. When OLM cells are driven by PYR input to a regime above spiking threshold without OLM-PYR feedback they have a tendency to fire slow gamma bursts around theta frequencies albeit fairly irregularly and incoherently across cells. When OLM-PYR feedback is included a PING-like reverberation emerges which interacts with the intrinsic OLM gamma activity to amplify it and consequently synchronizes the OLM cells at both gamma and theta frequencies. We refer to this synchronization mechanism as ‘PING-like’ because in true PING the cells do not need an intrinsic tendency to spike at gamma frequencies; gamma is created simply by the inhibitory-excitatory feedback. Nevertheless the inhibitory-excitatory interaction here has many similarities to PING. As in true PING, synchronization starts when one or more OLM cross threshold and spike, for example Fig 2C (red arrow). The resultant IPSPs in PYRs cause a few PYR to delay their subsequent spikes, creating a gamma frequency interval in the PYR spiking activity, after which several PYR spike together. This increase in synchronized PYR spiking causes more OLM to spike together. The spiking of PYRs becomes weakly synchronized and the spiking of OLMs also becomes synchronized due to the feedback between the two populations, although the populations have some phase difference, Fig 2I (blue, orange). Furthermore at the lower SCILs there are OLM cells which would be permanently subthreshold if subject to the same PYR drive in isolation, but now spike due to the focussed coherent PYR excitatory bursts generated by the PING feedback. Thus the PING burst not only synchronizes the OLM cells at gamma frequency but provides a ‘theta phase reset’ which also synchronizes them at the theta burst frequency. After several coherent population spikes the theta burst ends due to the intrinsic currents in OLM cells which promote burst firing. On the other hand when the SCIL is increased too high, above about SCIL 190 in the SM, intrinsic theta bursting does not occur in the OLM cells, Fig 5E (orange), and they become synchronized purely at gamma frequency by the PING mechanism. Thus population gamma power increases strongly above SCIL 190 in the SM, Fig 5F (blue), but only weakly in the SMx, Fig 5F (orange).

### Network generated PING-like reverberation also enhances theta at the single OLM cell level

We wondered if this theta enhancement could be explained purely by a PING driven synchronization of OLM cells which are already intrinsically regularly burst firing at theta frequency, abeit incoherently across cells, or alternatively if the PING-like reverberation was also causing individual OLM cells to burst fire at theta frequency more strongly and regularly. In the former case we would expect to find an effect of OLM-PYR feedback at the OLM population level but not at the OLM single cell level. We therefore investigated peak theta and gamma strength in individual cells. Fig 5H shows the peak OLM theta strength calculated from all individual cells before averaging across cells. We find OLM cell theta strength is significantly enhanced in the SM, Fig 5H (blue), compared to the SMx, Fig 5H (orange), although the enhancement is not quite as strong as found at the population level, Fig 5E (blue, orange). Thus, interestingly, as well as synchronizing the theta activity across the population, the PING-like feedback also enhances and regularizes theta bursting in individual cells. Less surprisingly, given the PING-like feedback mechanism, gamma activity in individual cells is also enhanced by OLM-PYR feedback in the SM, Fig 5I (blue) compared to the SMx, Fig 5I (orange).

### Theta frequency and strength also depend on PING characteristics

This suggests the interesting possibility that by changing the properties of the PING-like reverberation we can not only affect gamma frequency and strength, but, due to the coupling to the intrinsic OLM bursting mechanism, we can also affect the strength and frequency of the population theta. To confirm this we modified the PING mechanism. In PING the gamma frequency depends on the timescale of decay of IPSPs generated by the inhibitory cells in the excitatory ones [40, 42, 43, 68, 75]. Once an IPSP has decayed the excitatory cell is free to spike again. If the IPSP is larger, or has a longer synaptic decay timescale, the PING frequency is expected to decrease. If network feedback PING is interacting with an intrinsic gamma generated by the OLM cells then we expect that this interaction will not be possible if the PING gamma timescale is too long. If IPSPs are small a longer synaptic decay timescale will be acceptable. Indeed the FM simulations, Figs 2, 3 and 4 use a GABA decay timescale of 18 ms and a small synaptic strength estimated from the fit to data, Fig 1, while the SM, Fig 5(blue), uses the same synaptic strength but an 11.8 ms GABA decay timescale. Since the synaptic strength estimated from empirical data, Fig 1C, is small, a PING-like reverberation is possible despite these slow decay timescales.

To reduce the capacity for PING to interact with the OLM cells we therefore increased the IPSP decay timescale to 25 msec and also increased the synaptic strength threefold in a modified simplified model, denoted SM25. In this case TSGPAC magnitude, Fig 5G (green), theta, Fig 5E (green), and gamma, Fig 5F (green), strength are greatly weakened compared to the SM results, Fig 5G, 5E and 5F (blue). Weakened theta and gamma are also evident in the average OLM single cell power spectrum from the exemplar simulation of the SM25 at SCIL 178, Fig 6C2 (green), and in the PAC comodulogram, Fig 6C3, while the raster plot, Fig 6C1, and membrane potential trace, Fig 6C4, look similar in appearance to the SMx model without OLM-PYR feedback, Fig 6B1–6B4. This suggests that the amplification and synchronization of OLM theta does indeed occur through a PING-like feeback reverberation.

Finally we investigated what happens when the OLM GABA timescale is made much shorter, 7 ms, in the simplified model, denoted SM7, while still maintaining the threefold increase in OLM-PYR synaptic strength used in the SM25, so that PING should be strong. In this case, OLM TSGPAC magnitude, Fig 5G (red), OLM theta, Fig 5E (red), and OLM gamma, Fig 5F (red), strength are strongly increased compared to the SM25 results, Fig 5G, 5E and 5F (blue), as can also be seen in the exemplar OLM single cell power spectrum from the same simulation at SCIL 178, Fig 6D2. As expected the gamma frequency increases, Fig 5C (red), due to the shorter IPSP decay time. The theta frequency also decreases, Fig 5B (red), because theta bursts are now longer and consist of more gamma frequency spikes due to the stronger feedback PING as seen in the raster plot, Fig 6D1 (red), and membrane potential trace, Fig 6D4. Thus, interestingly, we find that parameters of network generated PING not only affect the gamma frequency and strength but, due to the interaction with OLM intrinsic bursting, also affect the theta frequency and strength.

The effects of OLM-PYR STP depression on theta and gamma described above in the FMfix, Fig 4(green), are consistent with this feedback PING-like reverberation. OLM cells can only intrinsically show the theta bursting behaviour when they are in an excitation window just above threshold. If OLM-PYR synapses are depressing the inhibition onto PYR cells successively decreases each OLM spike during an OLM theta burst, thereby reducing the feedback coupling between the OLM and PYR populations. If OLM-PYR depression is removed the coupling becomes much stronger and a permanent strong PING like state is established at lower SCIL, Fig 4I (green). Therefore OLM activity dependent OLM-PYR STP depression maintains the OLM cells closer to threshold up to higher SCIL making the theta bursting state much more robust across a wider range of SC driving. The reduction of gamma frequency which occurs when OLM-PYR depression is absent, Fig 4F (green), is also consistent with the PING-like mechanism because the stronger IPSPs generated take longer to decay. Without OLM-PYR depression PING coupling is stronger so theta bursts also increase in length and contain more gamma frequency spikes which acts to reduce the theta burst frequency, Fig 4E (green), in a similar way to described above for the shorter IPSP decay timescale in the simplified model, SM7, Fig 6D1 and 6D4.

### Dependence of theta on OLM cell ion channels

Several studies have investigated intrinsic theta generation in OLM cells. This has been thought to depend on the h-current [53, 55] although in *in-vivo* like depolarized states it has also been suggested that h-channels may not be relevant [54]. Interestingly Sekulić and Skinner [76] also found a strong involvement of the slow-delayed rectifier potassium current, K_DRS_, in OLM theta, which they found to work together with the I_h_ current. We therefore wondered if similar currents played a role in the theta periodic gamma bursting in the optimized data-driven OLM cell model we use here.

**Fig 7:**
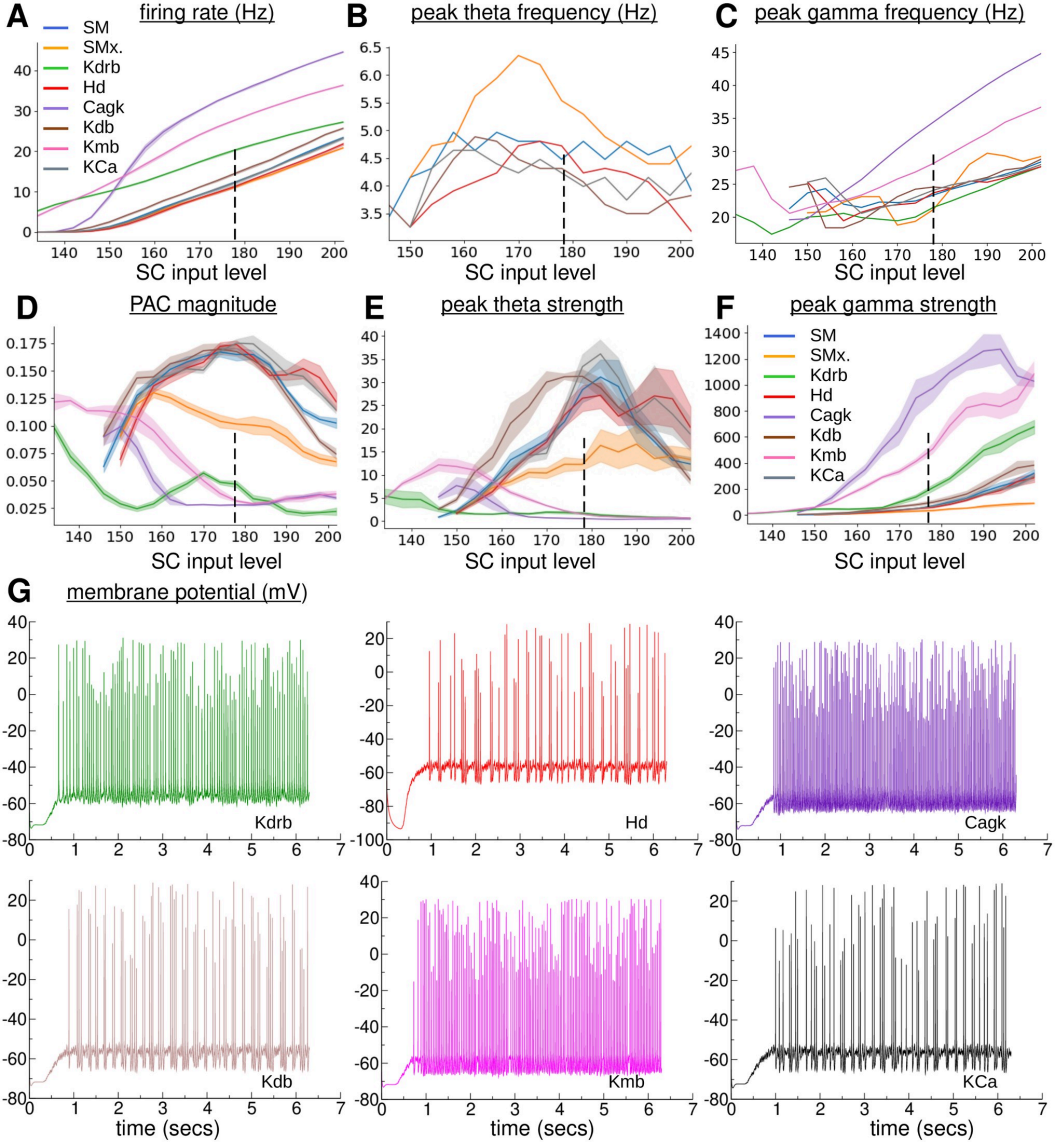
Dependence of TSGPAC on SCIL in the 120 PYR, 10 OLM SM model with OLM ion channel knock-out. *Blue: unmodified SM model. Orange: unmodified SMx model with OLM-PYR connections removed. Green: SM model with OLM* K_DR_ *knock-out. Red: SM model with OLM I_h_ knock-out. Purple: SM model with OLM Cagk knock-out. Brown: SM model with OLM* K_D_ *knock-out. Pink: SM model with OLM* K_M_ *knock-out. Grey: SM model with OLM* K_Ca_ *knock-out*. **(A)** *OLM firing rates*. **(B)** *OLM population peak theta frequency. Location of power spectrum peak in 1-8 Hz band*. **C** *OLM population peak gamma frequency. Location of power spectrum peak in 15-50 Hz band*. **(D)** *OLM population total PAC magnitude in the theta, 1-12 Hz, band and slow-gamma, 12-50 Hz, band*. **(E)** *OLM population theta strength. Theta band power spectrum peak size*. **(F)** *OLM population gamma strength. Gamma band power spectrum peak size*. **(G)** *Exemplar membrane potential traces from a cell in the simulations at SCIL 178, indicated by the dashed vertical line in (a-f), (see labels.)* **(A,D-F)** *Shaded bands show SEM in the plotted values across two simulations with random configurations. SEM not shown in (b,c) for clarity*.

The OLM cell model we utilise includes a complete set of active membrane properties [73]. Besides the nonspecific I_h_ current, they include a sodium current (Na), four types of potassium (K_DR_, K_A_, K_M_, and K_D_), three types of Calcium (CaN, CaL, CaT), and two types of Ca-dependent K+ currents, K_Ca_ and Cagk. A simple Calcium extrusion mechanism is also included in all compartments containing Calcium channels. Unlike hypothesis driven models which may include only minimal components of interest in order to specifically investigate their behaviour, the current network model, including the cell models, is entirely *data driven*. The OLM cell parameters, in particular the ion channel proportions, have been optimized by genetic algorithm [73] to capture many empirical features of the spiking and electrophysiology of OLM cells. We therefore expect to find degeneracy, whereby multiple ion channels may contribute to any given cell phenonena of interest, [73], and similarly any given ion channel may affect multiple distinct properties of interest. Therefore to investigate which of the active channels contribute to the preferential theta frequency spiking in the current OLM model we knocked-out individual ion channels one at a time by zeroing their maximal conductance parameters. In each case we performed a set of simulations of the SM model, Fig 5, at increasing SCIL. The results are shown in Fig 7.

One by one individual knock-out of I_h_, (Fig 7A–7G (red)), K_D_, (Fig 7A–7G (brown)), K_Ca_, (Fig 7A–7G (grey)), K_A_, (not shown), CaN, (not shown), CaL, (not shown), CaT, (not shown) did not produce strong effects on the TSGPAC properties of this network model, although the SCIL where OLM cells reach firing threshold does vary a little. Knock-outs of three ion channels, K_DR_, (Fig 7A–7G (green)), CagK, (Fig 7A–7G (purple)), and K_M_, (Fig 7A–7G (pink)) do seem to produce strong effects however.

Knock-out of the K_DR_ ion channel strongly reduces the OLM firing threshold, Fig 7A (green). Like [76] theta strength, Fig 7D and 7E (green) and TSGPAC magnitude are strongly reduced compared to the control model, Fig 7D and 7E (blue), although the OLM population does still show strong slow gamma, Fig 7F (green) with similar frequency to the control model, Fig 7C (green), and OLM firing rates do still increase slowly above threshold SCIL, Fig 7A (green), in a similar way to the control model, Fig 7A (blue). Knock-out of the CagK ion channel, Fig 7(purple), also strongly reduces theta strength, Fig 7E (purple) and TSGPAC, Fig 7D (purple), while strongly increasing gamma strength and frequency, Fig 7F and 7C (purple). In this case the OLM firing threshold is not affected, but the OLM firing rate increases much more rapidly, Fig 7A (purple), than the control model, Fig 7A (blue) as SCIL is increased initially, but more slowly at higher SCIL. The effects of K_M_ knock-out, Fig 7(pink), are somewhat different. In this case the OLM firing threshold is reduced to lower SCIL, Fig 7A (pink), and OLM firing rate increases a little more rapidly than in the control model, Fig 7A (blue). However some theta, Fig 7E (pink), and TSGPAC, Fig 7D (pink), is still found in the OLM population, albeit at lower SCIL.

A full investigation of why these channels are necessary for theta is outside the scope of the present paper. However K_DR_ is one of the potassium currents which is involved in control of the resting membrane potential, the threshold for action potential initiation, the action potential repolarization, and the after-hyperpolarization (AHP), determining the spike frequency and discharge characteristics of OLM [77]. K_DR_ knock-out affects the spike AHP necessary for slow oscillations. Cagk is a Ca and voltage-dependent K+ conductance. Its efficacy is determined by the internal [Ca]_*i*_ concentration, suggesting the involvement of Ca dynamics. Indeed we found that aside from CagK and K_DR_ at least one of the Ca channels, CaN, CaT, CaL is necessary for TSGPAC although any one could be knocked-out individually. The issues will be further addressed in future studies.

### Comparison with experimental findings

**Fig 8:**
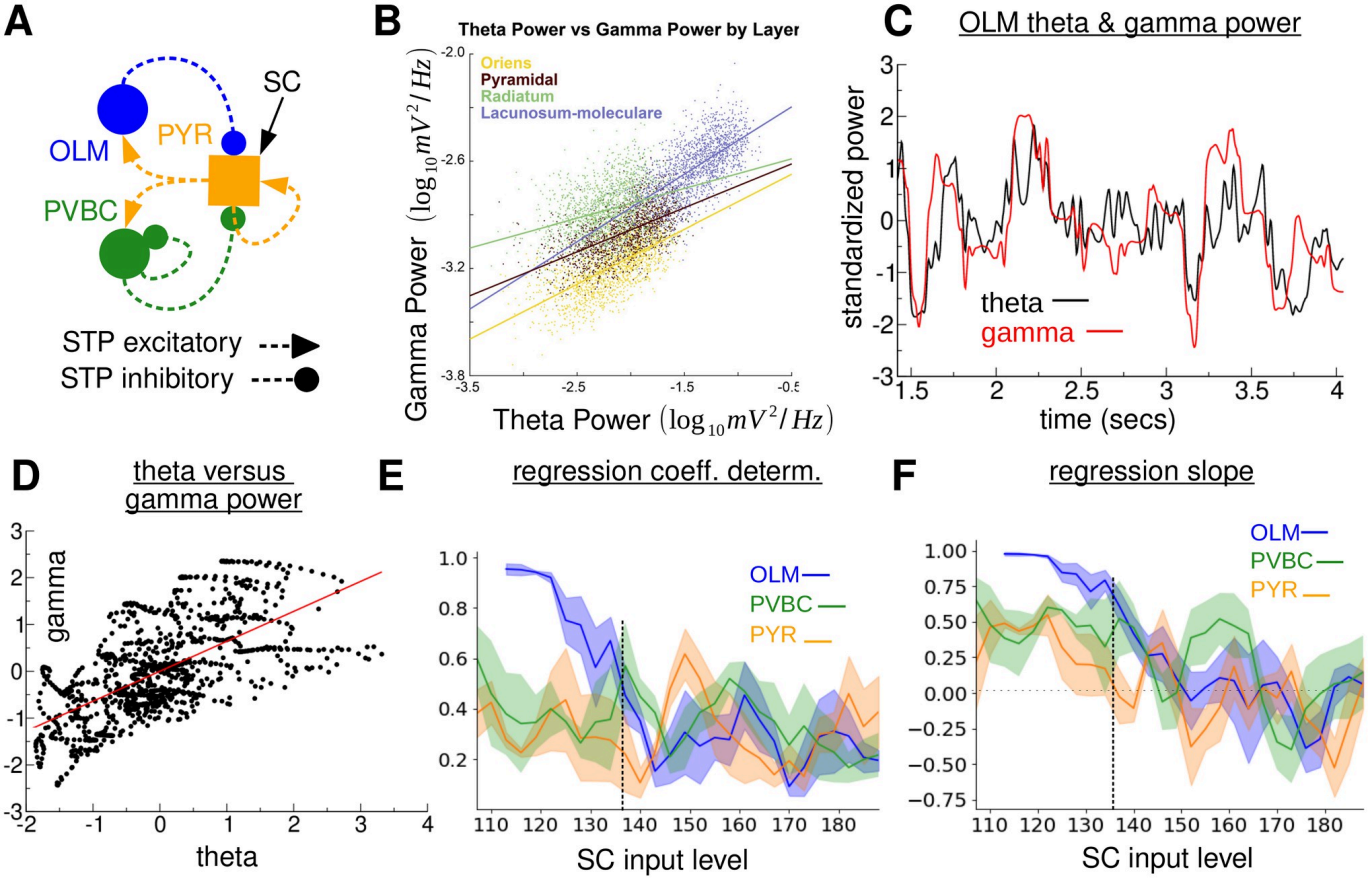
Theta and gamma power strongly covary in the 480 PYR, 20 OLM, 20 PVBC full model with facilitating/suppressing connections investigated in Figs 2 and 3. **(A)** *Circuit schema*. **(B)** *Empirical comparison. Scatter plot and least-squared regression lines for theta and gamma power across the layers of a rat. There is a clear positive trend across all layers, although it is strongest in the lacunosum-moleculare. Reproduced from* [57]. **(C)** *Time development of standardized theta (black) and gamma (red) power in a one second window centered on the plotted time point for the simulation described in* Fig 2. *Theta power is calculated in a 4 Hz band centered on the peak theta frequency (4.5 Hz) and gamma power in a 16 Hz band centered on the peak slow gamma frequency (25 Hz). Both time series are standardized*. **D** *Illustration of standardized theta power plotted against standardized gamma power (black) for the results in C and linear regression (red)*. **(E,F)** *Coefficient of determination E and slope F for linear regression of theta against gamma power for many microcircuit simulations at different levels of SC driving for the three cell populations. Error bands show SEM over two network simulations with identical parameters. Vertical dashed line indicates simulation in (b-d) at SCIL 136*.

In agreement with studies which suggest theta and gamma emerge as a unitary process [57], we found that theta and gamma frequencies interact. Gamma frequency and power [57, 78–82] as well as theta power [57, 81] and frequency [78] are known to increase with increasing running speed. If theta and gamma arise from distinct circuit and biophysical mechanisms then dissociable changes in power in the two bands should be found as synaptic drives change during different behaviours. On the contrary theta and gamma power are found to strongly covary, [57] (see Figure 5A), reproduced in Fig 8B. Other studies have also found covariance of theta and gamma cycle length [10] (see Figure 8B,C).

Indeed the OLM population activity spectrogram for the simulation of the FM, Fig 2H, suggests that theta and gamma power may covary in time. To investigate this in more detail we calculated theta and gamma power, Fig 8, in a moving one second window for the same simulation investigated in Fig 2. We found that the power of theta (black) and gamma (red) covary strongly with time, Fig 8C. This is due to slow deterministic rate variations generated by network feedback and because the input from the SC collaterals is stochastic spiking. A strong episode of PING can generate a strong episode of population theta by reverberating with the OLM intrinsic bursting mechanism. When PING is weaker, OLM cells spike more irregularly and less coherently across cells. Strong covariation between theta and gamma power was confirmed by linear regression of the simultaneous gamma power versus theta power, Fig 8D. We calculated the coefficient of determination (a measure of the strength of the dependency), Fig 8E, and slope of the linear regression, Fig 8F, as the SCIL was varied for the same full network simulations investigated in Fig 3. Strong covariation of theta and gamma power was found for the OLM cell population across the whole SCIL range, Fig 8E (blue), with positive slope, Fig 8F (blue), indicating that theta and gamma power increase together. PYR and PVBC cell populations also show quite strong positive relationships between theta and gamma power but only at lower SCIL, Fig 8E and 8F (orange, green). The covariation is stronger at lower SCIL for all three cell populations because OLM cells are on average closer to threshold, or just below threshold, and therefore extended periods of OLM silence stochastically occur separated by bouts of the theta/gamma state. This causes high covariance between theta and gamma power.

## Discussion

Here we presented a neural mechanism for the generation of theta-gamma PAC, suggesting that the population theta oscillation itself requires gamma PING close to firing threshold [83]. The dependence of the slow oscillation on the fast one may be relevant for the ubiquity of PAC in the brain. We found that when OLM cells were driven just above firing threshold by many spiking processes they had a weak tendency to fire irregular bursts of slow gamma frequency spikes around theta frequencies. When coupled to a PYR population an OLM spike could initiate a burst of feedback PING which synchronized the OLM cells at both slow gamma and theta frequencies and created a robust population oscillation. This was not purely a synchronization of already regularly but incoherently burst firing OLM cells but also involved amplification and regularization of theta and gamma in individual cells. In the absence of OLM-PYR synaptic depression TSGPAC only existed in a narrow window of SCIL since OLM cells only display this bursting behaviour when just above threshold. However when OLM-PYR synaptic depression was included PING excitation weakened on successive OLM spikes promoting bursting and allowing the TSGPAC state to continue up to higher SCIL. Thus robust PAC resulted from a complex interplay of intrinsic cellular properties, PING-like network feedback and STP.

The current model has strong similarities a with recent interesting work [51], where cells which intrinsically fire gamma frequency spikes in low (theta or delta) frequency bursts were coupled. The rhythms of individual cells were particularly susceptible to noise when the cells were isolated. However when they were coupled synchrony emerged which resulted in a robust population slow rhythm which was resilient to noise. One difference is that the cells were directly coupled by gap junctions while in the current model they are indirectly coupled by PYR cells. However in both studies the coupling acts to make incoherent and noisy activity synchronous and robust. The intrinsic cellular currents responsible for bursting are also different. Here we find OLM cells do have a weak intrinsic tendency to fire slow gamma bursts at theta frequency but both the theta and gamma frequencies are also determined by the nature of the PING coupling. In contrast to [51] we also investigate the role of STP and find that OLM-PYR depression is especially relevant for TSGPAC.

We found that when the OLM-PYR IPSP decay timescale was reduced, while maintaining the OLM-PYR synaptic strength, gamma and theta were strongly enhanced at both the individual OLM cell level and the OLM population level. On the other hand when the OLM-PYR IPSP decay timescale was too long theta did not occur at all. Several studies of the OLM-PYR IPSP decay timescale have found it to be rather long, of the order of 20 ms [72, 84]. Modeling studies have used 18 ms [71] or 11.8 ms [74]. In the latter case the synaptic time constant was scaled down to account for the fact that while empirical IPSC recordings were performed at the PYR soma [72], model synapses were located throughout the dendritic tree [74]. The authors scaled the dendritic synaptic time constants so that model somatic IPSPs matched those determined in experimental somatic recordings. Here we used an 18 ms time constant for the full model, (Figs 2 and 3), and fit the IPSPs to the empirical study by Pouille and Scanziani [69] to determine IPSP strengths, (Fig 1). OLM-PYR synaptic strengths we used were very close to the ones given in [70]. Because the IPSP strength was small the PING-like reverberation still occurred despite the 18 ms IPSP decay time and we found strong theta and TSGPAC, Fig 3. We also used the 11.8 ms timescale in the simplified model, Figs 5 and 6, with the same weak synaptic strength, to demonstrate that strong theta and TSGPAC also occur in this case.

This is a data-driven model. We used detailed multi-compartment cell models with reconstructed dendritic morphologies which had previously been optimized to fit various electrophysiological features [73] and connected them with short term plasticity at empirically determined dendritic locations with synaptic parameters also estimated against data [70]. This detailed modelling allowed us to accurately fit the dependence of IPSP sizes in different PYR dendritic locations on the number of alveus stimuli as well as the spiking probability in the OLM and PVBC cells observed in studies [69]. We found that the existence of PAC and the obtained theta frequency for certain ranges of SC input was dependent on the estimated STP parameters. This does not mean that PAC utilizing the same PING bursting mechanism cannot be generated with simpler cell models, but the detailed models used here provide greater confidence that such a mechanism can be found in the real CA1 and would allow quantitative predictions of how experimental manipulations might affect PAC. Furthermore the detailed data driven model presented here provides insight into which ingredients would be necessary to include in simpler models for further investigation.

We have not addressed in detail the role of PYR-OLM synaptic facilitation. The simplified models we investigated, Figs 5, 6 and 7 retained PYR-OLM synaptic facilitation. However we found that bursting PING generating PAC could also be found without such facilitation, although the PYR-OLM synaptic strength has to be strongly increased outside the physiological regime to compensate for the absence of facilitation. Preliminary investigations also indicate that the width of the SCIL window where PAC is found depends on the strength of PYR-OLM facilitation. Indeed we found TSGPAC only existed in a window where OLM firing rates were just above threshold. If SC excitation levels vary dynamically during behaviour PYR activity level dependent PYR-OLM facilitation may also act to maintain the OLMs in this window. Indeed we found that in the absence OLM-PYR synaptic depression the PAC window ended when the OLM firing rate reached a critical value around 20 Hz, Fig 3B and 3H. When OLM firing rate gets too high each spike generates a PING feedback and a permanent PING state is established. OLM-PYR synaptic depression prevented this by reducing the PING strength up to higher SCIL.

Several theories for PAC have been proposed [2, 30, 31]. Most (but not all [85], [51]) involve two interacting subsystems oscillating at different frequencies with different intrinsic timescales [32–36, 86], or a single fast system externally driven by a slow one [87–90]. Detailed CA1 models generating coupled oscillations [71] also include many components with different timescales. In most of these models the slow oscillation is generated without the fast one. The PAC mechanism presented here is different to these since the generation of the slow oscillation at the population level requires the fast one. [91] presented an interesting model where coherent theta is generated when cells with a subthreshold theta resonance are coupled, further combined with a PING and ING mechanisms to produce gamma. However the mechanisms were separate in that the coupling to produce coherent theta did not itself also generate gamma and thus somewhat different to the current model. Of course we do not mean to suggest that slow oscillations themselves cannot be generated by different means (for example [14, 36, 92–94]) or that PAC cannot be generated by different means. Indeed a recent model demonstrates PAC emergence in a completely different way using a spatial dimension [95]. Most likely there are numerous different ways PAC is generated in the brain. However our model does suggest that there may be experimentally observed types of PAC where fully blocking the fast oscillation will also abolish the associated slow one.

This model is in good agreement with many experimental studies of theta-slow gamma PAC. In particular it may provide insight into the experimental observations that theta and gamma power and frequency covary [10, 57], suggesting they arise from a single process. These observations cannot be easily explained by some other models of PAC. In our model the gamma frequency at which PAC occurs depends on the level of excitation needed for OLMs to be close to threshold. Our model therefore predicts that reducing the maximal conductance of PYR-OLM synapses will increase the PAC gamma frequency since stronger SC excitation will be needed to bring the OLMs to threshold.

Hippocampal theta is often found around 8 Hz *in-vivo* but there are also known to be at least two types of *in-vivo* hippocampal theta, low (3-7 Hz) and high (7-12 Hz) [14]. Social stimuli elicit high theta, and fearful stimuli elicit low theta [13]. Low theta has been associated with risk taking behaviour in behaving animals [15] and with immobility or lack of attention [12, 96]. In humans theta frequency has been found many times to be lower in general [97–100]. Strong low theta (2-3 Hz) gamma (30 Hz) PAC has been found in the hippocampus ([8], see Figure 2 and Figure 3A), strongly resembling our PAC results, Fig 2H.

*In vitro* theta can be low, for example 5 Hz [24] and 3-5 Hz [21]. A recent study [15] showed that continuous optogenetic activation of OLM cells in the ventral hippocampus induced low frequency (type 2) theta (around 4 to 7 Hz) while OLM inhibition decreased low frequency theta. Strikingly PYRs phase locked to the induced theta and OLM stimulation did not elicit theta at all when PYRs were inhibited. This strongly suggests a feedback OLM-PYR circuit is necessary for low frequency theta as in the current model. In further powerful support for the current model it was found that OLM stimulation also induced gamma oscillations which were phase amplitude coupled to the optogenetically induced theta. The induced gamma frequency was different to the existing gamma before light stimulation, demonstrating that OLM activation synergistically generates both phase coupled oscillations.

In another study theta-frequency optogenetic stimulation of PYRs in CA1 slices generated gamma oscillations which were phaseamplitude coupled to the induced theta [25]. The oscillations were synchronous across the whole examined region, dependent on both AMPA and GABA receptors and excitatory neuron firing slightly preceded interneuron firing within each gamma cycle, suggesting gamma was generated by network PING. Strikingly induced gamma power was found to depend on the frequency of the theta optogenetic stimulation. This indicates that the light was not simply periodically modulating gamma PING amplitude, but that PING amplitude had its own intrinsic theta timescale, in good agreement with the current model. Peak gamma power also occurred around 5 Hz theta, close to the frequency found in our model, Fig 2G. Also in agreement with the current model modulation of PV activity specifically at theta frequency was found to produce resonant PYR activity [101]. We expect that the current PYR-OLM circuit will resonate if modulated at theta frequency, which could also occur via PVBC modulation [101].

Firing rates in our model are determined by network PING and PAC dynamics. *In-vivo* PVBCs fire around 8 Hz in mice at rest and about 25 Hz running, while OLMs show slightly higher rates of 10 Hz and 30 Hz [59, 102]. In *in-vitro* studies OLMs spiked at theta frequencies [20, 53, 61–63] on roughly one in four gamma cycles [61] or about 1.7 spikes per theta burst [23] while PV cells fire about 3.2 spikes per burst [23]. PYR firing rates during theta are generally found to be sparser [11, 23, 61, 103] but can be as high as 50 Hz during optogenetic stimulation to generate theta nested gamma [26]. Moreover during cognitive processing, such as running with strong theta nested gamma, small subsets of PYR cells are activated sequentially by CA3 and EC input for short periods of several seconds as place fields with high rates around 30 Hz [60]. Here, in good agreement with these observations, when we activate PYR cells by sustained SC input to physiologically plausible rate, we find OLMs vary between 2 and 30 Hz across the theta cycle while PVBC cells also fire around 15 Hz, Fig 2E and 2I, and many OLMs only spike on one or two gamma events during a theta burst while PVBC cells spike two or three times, Fig 2D. Moreover PYR firing rate was lower than the gamma frequency in general. For example in the full model between SCIL 120 and 180, slow gamma frequency increases from about 20 to 32 Hz, Fig 3F, while PYR firing rate is lower, increasing from about 15 Hz to 25 Hz. Thus PYR cell activity is relatively sparse and individual PYR cells do not spike on every gamma cycle. Preliminary investigations of larger (but computationally intensive) 960 cell PYR networks indicate that PYR firing rate can be much sparser, of the order 10 Hz, while still maintaining strong TSGPAC with slow gamma frequency between 25 and 40 Hz.

OLMs are often thought to be responsible for CA1 theta [20, 32, 33, 53, 61, 62]. However their precise role has been puzzling [14, 93, 104]. The theta frequency in our model was affected by several factors, including intrinsic properties of the OLM cell, the level of activity of PYR cells presynaptic to the OLMs as well as the strength of OLM-PYR synaptic depression. It has been suggested that OLMs are intrinsic theta oscillators dependent on the h-current [53, 55] although in *in-vivo* like depolarized states it has also been suggested that h-channels may not be relevant [54]. Indeed we did not find a strong involvement of the I_h_ current in theta generation in the current OLM cell model. Nor did we find involvement of the D-current, K_D_, which has been found to be strongly involved in PAC in some studies [51]. We did however find a strong role for the delayed-rectifier potassium current, K_DR_, Fig 7B, which may be related to its ability to generate a spike AHP. We found that the intrinsic theta oscillation in OLM cells required a calcium-dependent potassium channel, Cagk, and at least one Ca channel. In a standard bursting framework [105] calcium entry during repetitive spiking in a burst slowly activates the outward potassium current and makes the neuron less excitable. Eventually due to this process the spiking stops, calcium is removed, the Cagk deactivates and the cell is ready to fire a new burst of spikes. An experimentally testable prediction of the model is that modification of the calcium decay timescale may alter the theta frequency. However we also found the potassium delayed rectifier, K_DR_, to be necessary. This current keeps the membrane more hyperpolarized after each spike. Interestingly potassium delayed rectifiers were found to be relevant for OLM theta in work by Sekulić and Skinner, [76], where they worked in tandem with I_h_ current, although we did not find a strong involvement of this latter current. Thus Cagk may be playing a role similar to I_h_ in the current cell model.

We were not able to make the OLM burst fire with current injection and burst firing was only found when the cell was in a fairly narrow regime just above threshold. This suggests that fluctuations generated by spiking may be interacting with the intrinsic calcium resonance when close to threshold, reminiscent of stochastic resonance. Indeed our preliminary observations to be addressed in future work indicate that intrinsic bursting is stronger when spiking generated fluctuations are smaller (but present), such as when PYR-OLM synaptic strengths are weaker and more numerous and when spiking inputs are distributed over the dendritic tree.

Some studies have found that OLMs do not generate theta when driven with unmodulated input [64] but they do respond preferentially to theta frequency modulation, [62, 64]. In agreement with these observations our results suggest that the weak intrinsic theta oscillatory properties of OLM cells when close to threshold, Fig 4I, may enable them to resonate if driven by theta frequency modulated synaptic input. However in order to produce a coherent population theta rhythm they need to be coupled via PYR cells, which themselves also need to be driven above firing threshold.

CA1 cell types display particular spiking phase relationships with theta [23, 24, 58–60, 102] which depend on the particular experimental setting. In behaving mice, PV cells fire on the descending phase of LFP theta, whereas SST neurons fire near the trough [58–60]. In contrast in-vitro both PV and SSTs activate together just before the peak of LFP theta [23]. We find that the firing phase of PVBC cells with respect to theta strongly depends on the PYR-PVBC synaptic strength. At the value used in Fig 2, PVBC activity slightly precedes OLM activity closer to the peak of PYR activity while OLM activity peaks on the descending phase of PYR activity. However if the PYR-PVBC conductance is increased 50%, we find PVBC activity more closely follows PYR activity. Thus the model predicts that a rather small change in the overall synaptic input, as may occur in in-vitro versus in-vivo models, can strongly affect theta phase relationships.

PV interneurons are often found to be the most strongly involved in gamma oscillations [24, 52–54, 59]. Indeed we found that PVBCs also generated strong PING with stronger gamma power than OLMs, Fig 3H, at lower SCIL and strong gamma at higher SCIL. Experimentally PVBCs are found to discharge a little before OLM cells [59] also as in the current model, Fig 2C and 2I. These effects occur because the PYR-PVBC connections are stronger than the PYR-OLM ones, which is also consistent with the recent observation that PV neurons receive much greater excitation from the network compared with SST neurons [23]. During 40 Hz gamma PVBC cells fired near the gamma peak while PYR cells were active during the trough and ascending phase, [24], again as shown by our model, Fig 2C, because PYR cells fire diffusely after decay of inhibition from the interneurons.

However, we also found that OLMs could generate slow gamma oscillations amplified by the PING reverberation mechanism. In agreement with this, the selective role for PV interneurons in gamma generation has been challenged by several studies in different brain areas. In CA1 multiple distinct theta nested gamma types are known, including a 30 Hz ‘apical tuft’ gamma which was found to be fully independent of both PVBCs and the 50 Hz ‘perisomatic’ gamma they are associated with [19]. In both CA1 and CA3, gamma oscillation amplitudes increase on the peak of the theta cycle [9, 11, 16, 106], whereas in contrast PVBC preferentially fire on the descending phase [58, 59, 102]. This is in agreement with the current model since gamma power varies with OLM activity. In hippocampal CA3 photoinhibition of SST rather than PV interneurons more easily disrupted slow-gamma and slow-gamma frequency was modulated more easily by manipulation of SST than PV interneurons [68]. In visual cortex SST interneurons were also strongly involved in gamma [66, 67]. In future work it will be interesting to investigate whether true PING gamma generated by PVBC-PYR feedback can interact with the PING-like amplification we found in the OLM-PYR circuit, for example by changing the strength of PING and thereby the strength or frequency of theta. Interestingly recent works have found that optogenetic stimulation of parvalbumin neurons at 40 Hz slow gamma (but not 80 Hz) restores hippocampal slow gamma amplitude, and theta slow gamma PAC in Alzheimer’s (AD) mice model [107]. The study found that restoration of slow gamma oscillations during memory retrieval rescued spatial memory. Theta is often found to be involved in cognitive memory intensive tasks. Our finding showing that enhanced gamma can amplify and synchronize cells at theta frequency may provide insight into how stimulation at gamma frequency can affect slower timescale memory retrieval processes with relevance in AD.

There are of course many interneuron types in CA1 which we have not included here. Prominent among them are IVY cells and bistratified cells [70] as well as VIP expressing cells [108]. We focussed on the OLM-PYR circuit because this has been strongly implicated in theta generation and because OLM cells do not strongly recurrently connect to each other allowing the extraction of a simple OLM-PYR feedback motif. We plan to include other types in future investigations.

## Conclusion

In conclusion, we proposed a mechanism for the generation of slow oscillations which intrinsically includes PAC emergence by periodically bursting PING. The model has shown that it can provide insight into several theta gamma PAC findings in the hippocampus. However, the mechanism is very general. We expect it can occur in other interneuron types and in other regions of the brain, if these cells have a feedback relationship with excitatory neurons, an ability to fire at low frequencies and if the synapses to excitatory cells show activity dependent depression.

## Materials and methods

A simple circuit, Fig 1A, including pyramidal cells, OLM cells and PVBC cells was constructed using synaptic parameters matching experimental recordings described in [70], and using morphologically detailed cell models whose spiking and dendritic properties have been optimized to match experimental recordings, [73]. Cell models contain most of the active membrane channels known to be expressed in CA1 cells. Single cell models were downloaded from the “live papers” section of the Cellular Level Simulation Platform of the EBRAINS Infrastructure (https://live-papers.brainsimulation.eu/#2018-migliore-et-al). We used model CA1_pyr_cACpyr_oh140807_A0_idH_20170915113422 for the pyramidal cells, CA1_int_cAC_011017HP2_20180121160005 for OLM cells, and CA1_int_cNAC_060314AM2_20180120154747 for PVBC cells. Synaptic properties and connectivity were implemented as determined in [70].

### Model validation

To reproduce [69] findings, OLM and PVBC cells were driven by a point processes modeling the connections from pyramidal cells axons activated by alveus stimuli. The alveus stimuli onto OLM and PVBC cells were mediated by deterministic PYR-OLM and PYR-PVBC Tsodyks-Markram (TM) facilitating and suppressing synapses [71, 109–111]. Peak synaptic conductances were chosen from a uniform random distribution in the range [0.2, 0.4] nS for PYR-OLM, and [0.3, 0.7] nS for PYR-PVBC connections. These are a bit smaller than the range estimated in [70]. In fact, providing the PYR-OLM and PYR-PVBC synaptic strengths remain in proportion, variations in their absolute values does not make a strong change in the model behaviour because the same level of excitation can be provided by varying the number and/or firing rate of the presynaptic PYR cells. PYR connections form 5 synapses on OLM cells and 6 synapses on PVBC cells. Alveus stimuli all have a 1 ms time delay.

For the calculation of spiking probability, Fig 1B, the strength of alveus stimuli was adjusted so that the threshold for spike generation was reached within the first four alveus stimuli between about 20% and 80% of trials, as described in [69]. This was obtained with 120 alveus stimuli for the OLM cell and 50 alveus stimuli for the PVBC cell. Alveus stimuli occurred at 50 Hz. Eighty trials for each cell type were performed with different random synaptic conductance parameters and synaptic locations to calculate spiking probability. We obtained a good fit to [69] by adjusting the parameters of TM synapses around the indicated ranges found in [70]. In particular PYR-OLM synapses are slowly facilitating and take the parameters, F, 470 ms, D, 38 ms, U, 0.07, *τ*_AMPA_, 1.7 ms, *τ*_NMDA_, 148.5 ms, NMDA ratio 0.28. PYR-PVBC synapses are rapidly depressing and take parameters are, F, 0 ms, D, 110 ms, U, 0.32, *τ*_AMPA_, 4.12 ms, *τ*_NMDA_, 298.75 ms, NMDA ratio 0.28. These parameters are fixed hereafter.

For the traces, Fig 1C, to provide a direct comparison with Fig.1b in [69], it should be noted that it is not clear in the experiments how many presynaptic interneurons are activated by a given alveus stimuli and what the proportion of interneuron to PYR synaptic failures are. Therefore we started from the values used in [70]. Peak OLM-PYR synaptic conductances were chosen uniformly distributed in the range [1, 1.4] nS which are close to those stated in [70]. Even so we needed to simultaneously activate three OLMs by the alveus stimulus to generate IPSPs of about the size observed experimentally [69]. However even when we only used one PVBC cell with the PVBC-PYR synaptic strengths given in [70], IPSP sizes were about twice as big as observed experimentally in [69]. We therefore decreased PVBC-PYR peak synaptic conductances to values uniformly distributed between [1, 1.2] nS. These values are about half the values in [70], but did result in IPSPs of the appropriate size, [69]. Fig 1C shows the result of a single simulation where the strength of alveus stimuli was adjusted so that the presynaptic PVBC cell spiked on the first two alveus stimuli and not the third, while the three OLM cells spiked only on the third stimulus. This required the OLM cells to be activated by alveus stimuli simulating 140 presynaptic PYR cells, while the PVBC cell was activated by alveus stimuli simulating 80 presynaptic PYR cells.

The PVBC-PYR and OLM-PYR synapses are also TM synapses with parameters close to those in [70]. OLM-PYR synapses are strongly depressing, with D, 1770 ms, F, 6 ms, and U, 0.3 and each OLM cell was connected to the PYR cell with 13 synapses, as estimated in [70], located randomly on the PYR distal apical dendrite further than 250 microns from the PYR soma. The OLM-PYR GABA decay time constant used in [70] seemed to be too small compared to experiment [72, 84] so we used the 18 ms value used in [71]. PVBC-PYR parameters are also strongly depressing [70], with D, 965 ms, F, 8.6 ms and U, 0.16, and each PVBC cell was connected to the PYR cell with 11 synapses, as estimated in [70], located at the soma or on the basal dendrite within 50 microns of the soma. The PVBC-PYR GABA decay time constant was 5.94 ms as used in [70].

For the calculation of ratio of the slope of the second IPSP to the first IPSP, Fig 1D, we used the same parameters and alveus stimuli strength as used for Fig 1C. In this case, the OLM cells should fire on the second alveus stimulus with fairly high probability while the PVBC cells should fire on the first stimulus with high probability. To reproduce this effect, we used three OLM and five PVBC cells. IPSP onset slope ratios, Fig 1D, are calculated at the soma and a given apical dendrite location. Slopes are obtained from the first 3 ms after IPSP onset.

As described in [69] the PYR is also current clamped at the soma and at five locations in the apical dendrites for the computations shown in Fig 1C and 1D. The soma current clamp has amplitude 0.3 nA and the dendritic ones, 0.01 nA. This results in a resting potential of about -60.5 mV at the soma, -62.1 mV at the apical dendrite, where Fig 1C was recorded, and -65.5 mV at the apical dendrite where Fig 1D, was recorded. These are slightly lower than those used in [69] however to increase them to the values used in [69] would cause the PYR to fire (albeit very slowly).

### Microcircuit network model

Here we connect the PYR, PVBC and OLM cells into a circuit respecting known physiology. All network simulations are performed using the NetPyNE simulation environment [112]. For the full model (FM) described in Figs 2, 3, 4 and 8 all synaptic parameters and synaptic locations for the PYR-OLM, PYR-PVBC, OLM-PYR and PVBC-PYR connections are as described above from the fit to [69] shown in Fig 1. PYR-PYR and PVBC-PVBC synaptic parameters are taken directly from [70]. PYR-PYR synapses are located on the apical dendrite between 100 and 350 microns from the soma on dendrites with diameter less than 2 microns, with peak synaptic conductances drawn from a uniform distribution of [0.5, 0.7] nS. For PVBC-PVBC, peak synaptic conductances are in the range [4.2, 4.8] nS.

[70] sampled neuron pairs at intersomatic distances of 0–200 *μm* to predict their connection probabilities and synapses per connection. Within this intersomatic distance they estimated roughly the following connection probabilites PYR-OLM, 0.35, PYR-PVBC, 0.35, OLM-PYR 0.4, PVBC-PYR, 0.25, PYR-PYR 0.35, PVBC-PVBC 0.35. They estimated roughly the following quantity of synapses per connection, PYR-OLM, 5, PYR-PVBC, 6, OLM-PYR 13 and PVBC-PYR, 11, PYR-PYR 3, PVBC-PVBC 5. According to [113] 92% of CA1 cells are PYR. Of the interneurons 4.3% are OLM and 14.4% are PV basket cells, [113]. Recent studies, [114–116] estimated the density of CA1 PYR to be 300,000 per mm^3^ in dorsal CA1 and 180,000 per mm^3^ in ventral CA1. In a spherical volume with radius 0.2 mm we therefore expect about 130 PV cells, 40 OLM cells and 10000 PYR. To simulate this large network we reduce the quantity of cells and increase the quantity of synaptic connections in proportion. We also consider cells to connected in an all-to-all fashion with a binomial distribution of synaptic contacts, each synaptic contact being formed with a probability given by the cell-cell connection probability. Indeed in a small network without distance dependent connectivity it is unlikely that one cell would receive a large number of synapses while a neighbouring cell would receive zero. For the full model (FM) described in Figs 2, 3, 4 and 8 for PVBC cells we used 20 groups, each group representing 4 cells. Thus each of the 20 PVBC model cells is connected to each of the PYR cells with a quantity of synapses which is a binomially distributed random number, *B*(*N*, *p*), with *N* = 4 × 11 and *p* = 0.25. Similarly each PVBC cell is connected to every other PVBC cell with a binomially distributed random number of synapses, *B*(*N*, *p*), where *N* = 4 × 5 and *p* = 0.35. All the connections for other cell types are formed the same way using the connection probabilities and synapses per connection as described above. For OLM cells we use 20 groups of 1 cell. It turns out that 10000 activated PYR cells is far too many; the interneurons are drastically over-excited. The number of PYR we use is constrained by computational cost. We can maintain the same level of drive to the interneurons by increasing their firing rate and reducing their number. In fact we find that 480 PYR cells firing at about 20 Hz generates realistic firing rates in the interneurons. Trial simulations using 960 PYR at around 10 Hz appear to produce similar network behaviour but were too computationally intensive to be pursued. Therefore in the full model for PYR cells we use 480 groups of one cell. The remaining 10000—480 PYR cells which should be included are considered to be below threshold or firing extremely slowly and therefore not relevant to the dynamics of the network. Indeed it is well known that PYRs are not all activated together by CA3 and entorhinal cortex. In fact small subsets may for example be activated discretely in tasks as place cells, [60], by different sensory cues, or proprioceptive feedback or different presynaptic grid cell combinations etc. Small subsets may also be activated during experimental protocols such as optogenetic stimulation. This full model investigated in Figs 2, 3, 4 and 8 represents the behaviour of a small PVBC, OLM microcircuit network when 480 of their 10000 presynaptic PYRs are activated to fire around 10 to 20 Hz. In the FMx, Fig 4, OLM-PYR connections are removed and in the FMfix, Fig 4, OLM-PYR STP is removed so the F and D parameters are set to zero.

The microcircuit is driven by Schaffer collateral (SC) inputs to the PYR cells. Each PYR cell is fully independent and receives multiple independent SC inputs. These are modeled as poisson spiking processes at 1.4 Hz, a rate taken from CA3 recordings. However to make simulations faster we combine poisson processes into a groups of 20, resulting in a rate of 34 Hz for each one. Therefore where SC excitation level is quoted as ‘180’, for example, there are in fact 9 poisson processes each with rate 34 Hz. Each one of these makes six synapses on a PYR cells within the SC dendritic range. SC inputs to PYR are located on the apical dendrite between 100 and 350 microns from the soma on dendrites with diameter less than 2 microns. Synaptic parameters are the same as PYR-PYR synapses [70] (*τ*_*NMDA*_ = 148.5 ms, *τ*_*AMPA*_ = 3 ms, NMDA-ratio = 1.22), except that there is no facilitation or depression. SC synaptic delays are randomly chosen from the range [0.5,2] ms. Peak synaptic conductances are uniformy distributed in [0.55, 0.65] nS. Increasing excitatory driving is modeled as an increasing number of SC inputs. There are two simulations with identical parameters but different random configurations at each level of SC input in Fig 3. Simulations are run for 6.3 seconds.

The simplied models, SM, Figs 5, 6 and 7, to illustrate the mechanism behind PAC, are identical to the FM except that only PYR and OLM cells are included and only feedforward PYR-OLM and feedback OLM-PYR connections are included, without any recurrent connections. To reduce computational cost instead of 480 groups of one PYR cells we have 120 groups of four PYR cells and instead of 20 groups of one OLM we have 10 groups of two OLM cells. PYR-OLM synaptic facilitation is included as in the full circuit model but STP is removed on the OLM-PYR synapses so their TM synaptic parameters F and D are set to zero. To make PING feedback stronger the OLM-PYR GABA decay time constant is also reduced to 11.8 ms, which is the value used in [74]. In the SMx OLM-PYR connections are removed. In the SM25 the OLM-PYR synaptic strengths are made three times those of the FM and the GABA decay time constant is increased to 25 ms. In the SM7 the OLM-PYR synaptic strengths are again made three times those of the FM but the GABA decay time constant is decreased to 7 ms. For each of these SM models, there are two simulations at each level of SCIL input with different random configurations. Simulations are run for 6.3 seconds. In the simulations with knocked-out ion channels we simply set the relevant peak synaptic conductance parameter to zero.

### Data analysis

Data analysis was performed using Python including packages SciPy, NumPy and PacTools. Simulations had length 6.3 seconds and the initial 2 second equilbriation time was discarded from analysis. Spike times are converted to PYR, PVBC and OLM cell population rate time series using 4 ms bins. Fig 2E show such rate time series further smoothed with a 30 point moving average. Power spectra, are calculated from the rate time series using the SciPy package, signal.welch, with a hanning window and 2048 ms windows. They are smoothed with a two point moving average. PAC plots, Figs 2G and 6 are calculated from the cell population rate time series from a single simulation using the pactools comodulogram package with the method, ‘penny’ [117]. TSGPAC magnitude shown in Figs 3, 4, 5 and 7, is the average PAC in the phase frequency, *f*_*θ*_, range 1–8 Hz, and amplitude frequency, *f*_*γ*_, range 15–50 Hz. The PAC peak value in Figs 3 and 4 is the maximum value of PAC found in this same frequency range. Peak theta and gamma frequencies in Figs 3, 4, 5 and Fig 7 are the locations of the maximum values found in the theta, 1-8 Hz and gamma, 15-50 Hz ranges from the power spectrum for the cell population firing rate activities, smoothed with a two point moving average. Theta and gamma strengths are the values of these maxima. When theta and gamma magnitudes are calculated from single cell activities, Fig 5H and 5I, power spectra are calculated from each individual cell’s rate time series separately, then smoothed with a two point moving average and the peak strength in the relevant frequency range found. This quantity is then averaged over all cells in the simulation. All figures where PAC magnitudes, PAC peak values, theta and gamma peak magnitudes and values are shown versus SCIL are averages across two randomly configured simulations with identical parameters and the shading shows SEM. These are further smoothed with a three point moving average across neighbouring SCIL.

To calculate theta averaged activity, Fig 2I, we follow a method similar to that employed in [16]. The OLM population theta peak frequency, *f*_Θ_, is determined for a model simulation. The OLM population activity is band passed in a 2 Hz width band centered on this peak frequency using a 5th order Butterworth filter from the scipy signal package. Time points of local troughs in the band passed activity are determined. Firing rate time series are cut into segments between successive troughs of the OLM theta. Segments with length smaller than (1 − 0.1)/*f*_Θ_ or larger than (1 + 0.1)/*f*_Θ_ are discarded. From the remaining segments the segment with the minimum time length is found. Using a moving average the larger segments are rescaled down to have the same length as the minimum segment. These segments are then averaged.

Spectrograms, Fig 2H, of the OLM population activity are obtained from their population firing rates using the scipy signal spectrogram package using a 256 ms window. To obtain results in Fig 8 first peak theta and slow gamma frequencies are obtained for each simulation. The spectrogram for each cell type population activity time series in each simulation is also calculated as above. Spectral power time series are extracted by averaging the spectrogram in a 1 Hz band centered on the peak theta frequency and a 8 Hz band centered on the peak slow gamma frequency. These time series are standardized by subtracting their means and dividing by their standard deviations. Finally theta and slow gamma power at each time point are linearly regressed and the coefficient of determination and slope found. Fig 8E and 8F show averages and SEM over two simulations with identical parameters at different levels of SCIL excitatory driving. The results in Fig 8C and 8D pertain to the single simulation also described in Fig 2.
